# Distinctive molecular features of radiation-induced thyroid cancers

**DOI:** 10.1126/sciadv.adw7680

**Published:** 2025-08-22

**Authors:** Danielle M. Karyadi, Tetiana I. Bogdanova, Cato M. Milder, Stephen W. Hartley, Olivia W. Lee, Michael Dean, Vladimir Drozdovitch, Elizabeth K. Cahoon, Sergii Masiuk, Mykola Chepurny, Liudmyla Yu Zurnadzhy, Vibha Vij, Cari M. Kitahara, Gerry A. Thomas, Gayle E. Woloschak, Dale A. Ramsden, Mykola D. Tronko, Stephen J. Chanock, Lindsay M. Morton

**Affiliations:** ^1^ Laboratory of Genetic Susceptibility, Division of Cancer Epidemiology and Genetics, National Cancer Institute, National Institutes of Health, Bethesda, MD, USA.; ^2^ Laboratory of Morphology of the Endocrine System, V.P. Komisarenko Institute of Endocrinology and Metabolism, National Academy of Medical Sciences of Ukraine, Kyiv, Ukraine.; ^3^ Radiation Epidemiology Branch, Division of Cancer Epidemiology and Genetics, National Cancer Institute, National Institutes of Health, Bethesda, MD, USA.; ^4^ Laboratory of Translational Genomics, Division of Cancer Epidemiology and Genetics, National Cancer Institute, National Institutes of Health, Bethesda, MD, USA.; ^5^ Radiological Protection Laboratory, State Institution National Research Center for Radiation Medicine Hematology and Oncology, National Academy of Medical Sciences of Ukraine, Kyiv, Ukraine.; ^6^ Department of Surgery and Cancer, Imperial College London, Charing Cross Hospital, London, UK.; ^7^ Feinberg School of Medicine, Northwestern University, Chicago, IL, USA.; ^8^ Department of Biochemistry and Biophysics, Lineberger Comprehensive Cancer Center, University of North Carolina at Chapel Hill, Chapel Hill, NC, USA.; ^9^ Department of Fundamental and Applied Problems of Endocrinology, V.P. Komisarenko Institute of Endocrinology and Metabolism, National Academy of Medical Sciences of Ukraine, Kyiv, Ukraine.

## Abstract

Papillary thyroid carcinoma (PTC) incidence increased after childhood exposure to radioactive fallout from the Chornobyl accident. We investigated PTC genomic profiles to distinguish radiation-induced versus sporadic oncogenic drivers by modeling dose and molecular characteristics by driver category: *BRAF^V600E^* (*n* = 132), RAS mutation (*n* = 31), fusions generated from two breakpoints and <20 base pairs (bp) breakpoint gain/loss (Fusion^2B<20bp^; *n* = 63), or ≥3 breakpoints and ≥1000 bp breakpoint loss (*n* = 20). The frequency of Fusion^2B<20bp^-PTC increased with increasing thyroid radiation dose, whereas all others declined. Clonal small deletion counts increased with increasing radiation dose for Fusion^2B<20bp^-PTC (*P* = 5.1 × 10^−4^) but not other drivers (*P* > 0.08). Clonal clock mutational signatures, marking the age of tumor initiation, were associated with age at the accident for Fusion^2B<20bp^-PTC (*P* = 8.2 × 10^−4^) but not other drivers (*P* > 0.21). Together, these results support a causal role for ionizing radiation in Fusion^2B<20bp^-PTC as a group but not other drivers.

## Teaser

Whole-genome sequencing reveals that only certain fusion-driven PTCs were likely caused by radiation exposure after Chornobyl.

## INTRODUCTION

Ionizing radiation exposure is a well-established risk factor for cancer (*1*), although the magnitude of risk varies by dose and tissue type (*2*). Decades of research have shown that DNA double-strand breaks (DSBs) are the most consequential form of ionizing radiation–related DNA damage. Evidence of DNA DSBs and their subsequent repair are most often identified as small deletions and structural variation (SV). Recent advances in next-generation sequencing technologies have enabled comprehensive genomic landscape analyses that allow for quantification of these genomic features. Ionizing radiation exposure has consistently been found to increase the frequency of small deletions and SVs, but not any type of single-base substitutions (SBSs), in both normal (*3*) and tumor (*4*–*6*) tissues. However, small deletions and SVs are not unique biomarkers of radiation exposure because they also can be caused by endogenous processes and other exogenous exposures, such as cigarette smoking and ultraviolet radiation.

The aim of the current study is to distinguish radiation-induced from sporadic oncogenic drivers in a recently examined set of papillary thyroid carcinomas (PTCs; *n* = 285) collected after childhood exposure to radioactive fallout from the Chornobyl nuclear power plant accident (*4*). We used two comparison groups of individuals without known radiation exposure: *n* = 70 unexposed PTC cases from the same regions in Ukraine born >9 months after the accident (*4*) and *n* = 69 PTCs with fusion/SV driven PTC from The Cancer Genome Atlas (TCGA), the only other published large-scale study of PTCs with whole-genome sequencing (WGS) data (*7*). Assuming that the PTC driver is the initiating event in carcinogenesis, we used WGS data to characterize each tumor by the pattern of DNA damage that generated the drivers, which were identified previously (Table 1). Specifically, we characterized fusion/SV drivers by the number of breakpoints and number of base pairs (bp) gained/lost at each breakpoint, which are indications of DNA repair mechanisms and efficiency because healthy cells engage efficient DNA DSB repair without any delays or loss of major DNA fragments (*3*, *8*, *9*). In addition, we characterized mutations as single base or dinucleotide substitutions, deletions, or insertions.

**Table 1.** Distribution of PTCs by the pattern of DNA damage that generated the driver. Alternative end-joining (alt-EJ), base pair (bp), DNA double-strand break (DNA DSB), nonhomologous end-joining (NHEJ), papillary thyroid carcinoma (PTC), single-strand annealing (SSA), structural variant (SV), The Cancer Genome Atlas (TCGA), and whole-genome sequencing (WGS).

			Chornobyl Tissue Bank^*^							TCGA (*n* = 69)^†^	
Driver type		Comments	Total (*n* = 355)		^131^I-exposed (*n* = 285)		^131^I-unexposed (*n* = 70)				
Detailed classification			*n*	(%)^‡^	*n*	(%)^‡^	*n*	(%)^‡^		*n*	(%)^‡^
**Fusion/SV driver**			**140**	**(100.0%)**	**113**	**(100.0%)**	**27**	**(100.0%)**		**69**	**(100.0%)**
**# Breaks**	**Gain/loss at the breakpoint**										
2	<20 bp at both breakpoints	DNA DSBs, enriched for repair by NHEJ or alt-EJ without delay in repair; no DNA fragment loss	66	(47.1%)	63	(55.8%)	3	(11.1%)		17	(24.6%)
2	<1000 bp at both breakpoints (20–999 bp at ≥1 breakpoint)	DNA DSBs; unknown contribution of repair mechanisms (NHEJ, alt-EJ, SSA) with delay in repair and/or DNA fragment loss	14	(10.0%)	11	(9.7%)	3	(11.1%)		13	(18.8%)
2	≥1000 bp loss at ≥1 breakpoint	DNA DSBs; unknown contribution of repair mechanisms (NHEJ, alt-EJ, and SSA) and potential for long delay in repair and/or DNA fragment loss	4	(2.9%)	3	(2.7%)	1	(3.7%)		3	(4.3%)
≥3	<20 bp at all breakpoints	DNA DSBs, enriched for repair by NHEJ or alt-EJ without delay in repair; no DNA fragment loss	1	(0.7%)	1	(0.9%)	0			1	(1.4%)
≥3	<1000 bp at all breakpoints (20–999 bp at ≥1 breakpoint)	DNA DSBs; unknown contribution of repair mechanisms (NHEJ, alt-EJ, and SSA) with delay in repair and/or DNA fragment loss	7	(5.0%)	6	(5.3%)	1	(3.7%)		7	(10.1%)
≥3	≥1000 bp loss at ≥1 breakpoint	DNA DSBs; unknown contribution of repair mechanisms (NHEJ, alt-EJ, SSA) and potential for long delay in repair and/or DNA fragment loss	32	(22.9%)	20	(17.7%)	12	(44.4%)		24	(34.8%)
**Other special categories**											
Large deletion (≥1000 bp)		DNA DSBs	6	(4.3%)	3	(2.7%)	3	(11.1%)		1	(1.4%)
Large duplication (≥1000 bp; not tandem)		DNA DSBs; microhomology-mediated break-induced replication	3	(2.1%)	0		3	(11.1%)		1	(1.4%)
Tandem duplication (≥1000 bp)		DNA DSBs	1	(0.7%)	0		1	(3.7%)		2	(2.9%)
Templated insertion		DNA DSBs; microhomology-mediated break-induced replication	3	(2.1%)	3	(2.7%)	0			0	
Dicentric		DNA DSBs; repaired chromosome resulted in two centromeres	2	(1.4%)	2	(1.8%)	0			0	
Chromothripsis		DNA DSBs	1	(0.7%)	1	(0.9%)	0			0	
**Mutation driver**			215	(100.0%)	172	(100.0%)	43	(100.0%)			
SBS			200	(93.0%)	163	(94.8%)	37	(86.0%)			
Dinucleotide substitution			7	(3.3%)	5	(2.9%)	2	(4.7%)			
Deletion		Potentially caused by DNA DSB repair	1	(0.5%)	1	(0.6%)	0				
Insertion		Potentially caused by DNA DSB repair	1	(0.5%)	1	(0.6%)	0				
Multiple mutations			6	(2.8%)	2	(1.8%)	4	(9.3%)			

*Samples from the Chornobyl study eligible for this analysis were restricted to those with available WGS data, high tumor purity (>20%), and no evidence of tumor contamination in the normal tissue; we also excluded one tumor that did not have a known PTC driver, resulting in a total of 355 PTCs.

† Samples from the TCGA study eligible for this analysis were restricted to those with known fusion/SV drivers, available WGS data, and no known prior radiation exposure, resulting in a total of 69 PTCs.

‡ Column percent for fusion/SV and mutation drivers separately.

**Fig. 1.**
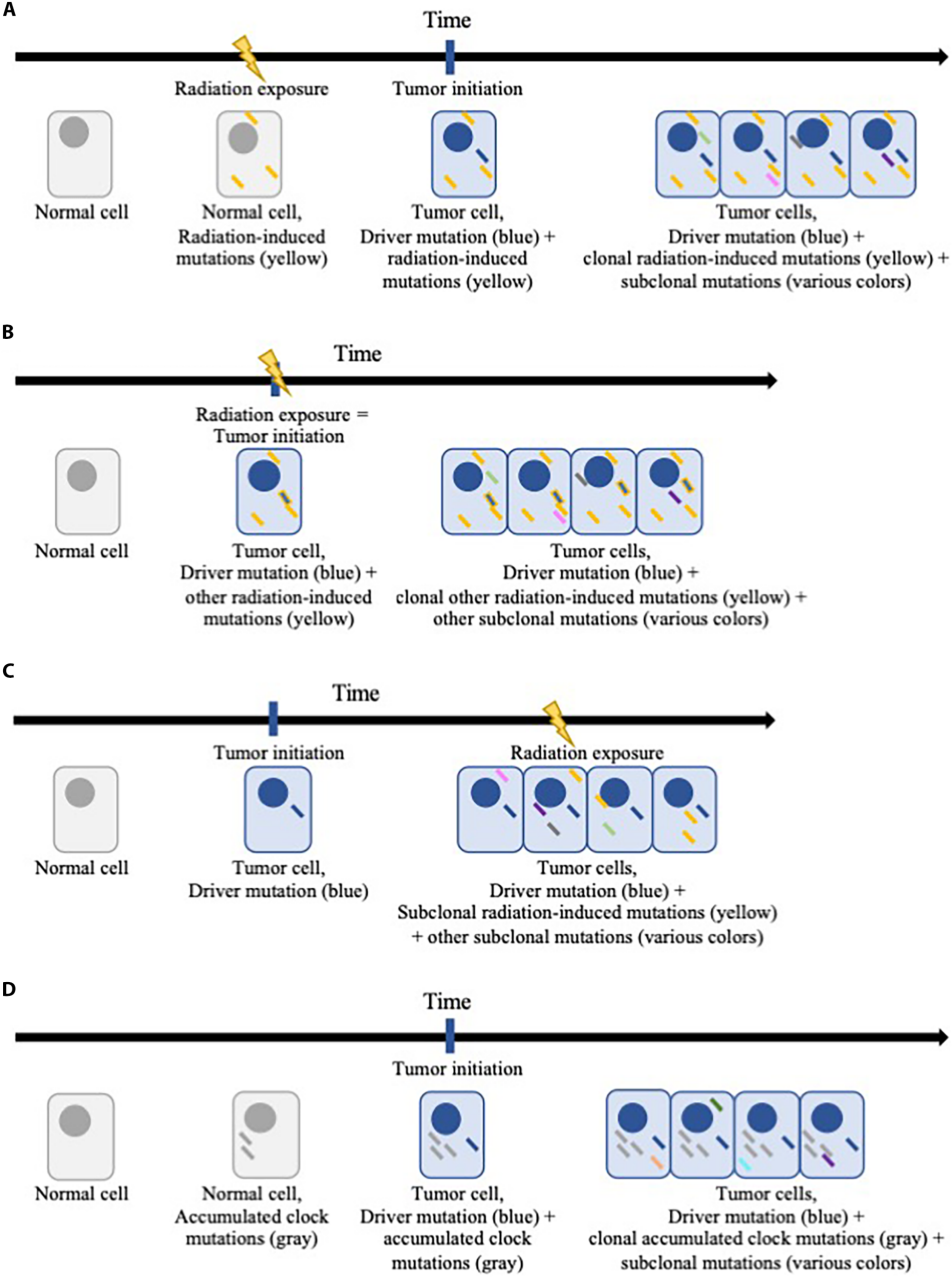
Schematics of mutation accumulation in normal and tumor cells. Accumulation of radiation-induced mutations under scenarios where radiation exposure occurred before tumor initiation (**A**), caused the tumor (**B**), or occurred after tumor initiation (**C**). Accumulation of clock mutations (**D**).

We then conducted a series of orthogonal analyses by driver category in exposed individuals to determine whether the tumors were consistent with having been caused by radiation exposure from the accident. First, we quantified the frequency of drivers by radiation dose, hypothesizing that radiation-induced tumors would be more frequent than sporadic tumors after higher-dose exposure. Second, we assessed the relationship between radiation dose and the clonal deletion:single nucleotide variant (SNV) ratio, which measures the rate of small deletions with adjustment for the background mutational rate in the tumor, hypothesizing that radiation-induced but not sporadic tumors would exhibit a positive dose-response relationship between radiation exposure and clonal deletion:SNV ratio (Fig. 1, A to C). Third, we estimated the timing of tumor initiation based on clonal clock SBS mutational signatures, which represent the mutations that had accumulated with age at the time of tumor initiation (*10*–*12*), hypothesizing that radiation-induced but not sporadic tumors would exhibit a positive relationship between clonal SBS clock mutations and age at the time of the Chornobyl accident (Fig. 1D).

The ability to distinguish between radiation-induced and sporadic cancers is of both public health and clinical importance in light of the spectrum of cancer risk people experience across a range of radiation exposures (*13*). Such distinctions have potential to improve understanding of the true consequences of nuclear accidents such as Chornobyl and Fukushima (*14*, *15*), inform optimal timing of thyroid cancer screening as well as compensation programs for individuals who develop cancer after radiation exposures (*16*, *17*), and lead to new insights into human tumorigenesis following ionizing radiation exposure and potential cancer prevention approaches.

## RESULTS

### PTC driver characterization

PTC drivers in the Chornobyl study and TCGA were previously identified (*4*, *7*) on the basis of recurrent mutations and fusions in each dataset and compared with COSMIC Cancer Gene Census v90 (https://cancer.sanger.ac.uk/census). We further characterized each tumor by the pattern of DNA damage that generated the driver. Among 285 PTC tumors in exposed individuals with available high-quality WGS data from the Chornobyl study, 113 (39.6%) had fusion/SV drivers and 172 (60.4%) had mutation drivers (Table 1). Seventy-seven (68.1%) PTC with fusion/SV drivers were generated from two DNA DSBs, most commonly with <20 bp of gain or loss at both breakpoints (Fusion^2B<20bp^; *n* = 63; fig. S1), whereas 27 (23.9%) were generated from ≥3 DNA DSBs, most of which had at least one breakpoint with ≥1000 bp loss (Fusion^≥3B≥1000bp^; *n* = 20), and 9 (8.0%) represented other special categories (e.g., large deletion, large duplication, and templated insertion). This distribution differed significantly from both unexposed comparison groups (*P*_Chornobyl_ = 1.9 × 10^−5^ and *P*_TCGA_ = 4.1 × 10^−4^): Fusion^2B<20bp^-driven PTCs were more common among exposed individuals (55.8%) than unexposed individuals in Chornobyl (11.1%) and unexposed individuals in TCGA (24.6%), whereas Fusion^≥3B≥1000bp^-driven PTCs were less common among exposed individuals (17.7%) than unexposed individuals (Chornobyl = 44.4%; TCGA = 34.8%) (Table 1).

The patterns of DNA damage that generated the fusion/SV drivers also differed by driver gene fusion partner and exposure group. Among exposed individuals, Fusion^2B<20bp^-driven PTC predominated (≥75.0%) for *NCOA4-RET*, *AGK-BRAF*, *BRAF-SND1*, *NTRK1-TPR*, *NTRK1-TPM3*, and *CREB3L2-PPARG* fusions, whereas the patterns were more variable for other *RET*, *BRAF* and all *NTRK3* fusions (table S1). In contrast, among unexposed individuals in both Chornobyl and TCGA, Fusion^2B<20bp^-driven PTC did not predominate for any specific driver gene fusion group (tables S1 and S2). Regardless of the driver gene, among exposed individuals, two-thirds (*n* = 42 of 63, 66.7%) of the Fusion^2B<20bp^-driven PTCs were inversions, whereas translocations were predominant among fusions with ≥20 bp of gain or loss at both breakpoints (<1000 bp: *n* = 9 of 11, 81.8%; ≥1000 bp: *n* = 3 of 3, 100%) (table S3). Inversions and translocations were more evenly distributed in unexposed individuals in both Chornobyl and TCGA for fusions generated by two DNA DSBs, regardless of the amount of gain or loss at both breakpoints.

In the 215 PTC with mutation drivers from Chornobyl, 200 (93.0%) were SBSs, most commonly *BRAF^V600E^* (*n* = 162) (Table 1 and table S1). The three *RAS* genes (*HRAS*, *KRAS*, and *NRAS*) were the next most frequently mutated (*n* = 39), with 32 SBSs and 7 dinucleotide substitutions. Although small indels can be caused by DNA DSB repair, we only observed two such single mutation drivers, both in *BRAF* (non-V600E), precluding further analysis.

Subsequent analyses focused on the two largest groups in radiation-exposed individuals from Chornobyl, *BRAF^V600E^* (*n* = 132) and Fusion^2B<20bp^-driven PTC (*n* = 63), with exploratory analyses of the next two smaller groups, *RAS* mutations (*n* = 31) and Fusion^≥3B≥1000bp^-driven PTC (*n* = 20). For Fusion^2B<20bp^-driven PTC, additional exploratory analyses separated inversions and translocations involving *RET* versus other thyroid oncogenes because these factors have previously been reported to be associated with radiation exposure (*5*, *6*, *18*).

### Radiation dose distribution

**Fig. 2.**
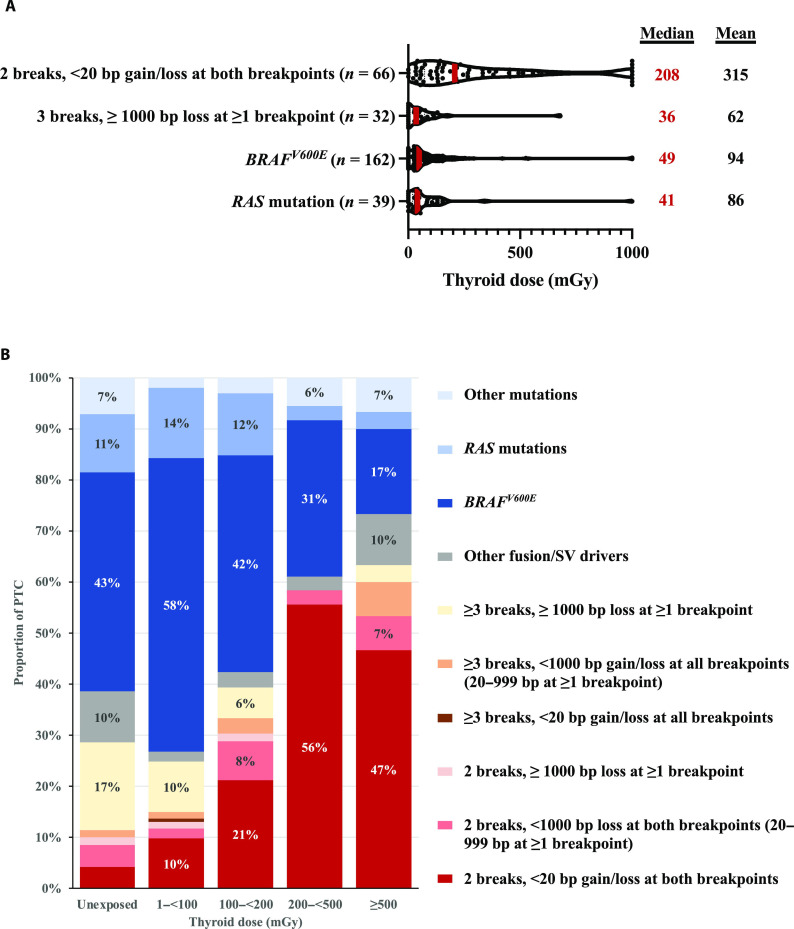
Distribution of thyroid radiation dose by the pattern of DNA damage that generated the PTC driver. Continuous dose distribution truncated at 1000 mGy for primary driver categories (**A**) and categorical dose distribution for all driver categories, showing percentages ≥5% (**B**).

Because PTC risk increases with increasing radiation dose (*19*), we hypothesized that radiation-induced tumors would be more frequent after higher dose exposure. The highest mean thyroid doses occurred in individuals with Fusion^2B<20bp^-driven PTC (*P*_heterogeneity_ among the four driver groups = 3.5 × 10^−5^, after adjusting for sex and age at PTC diagnosis) (Fig. 2A). Doses were higher for inversions than translocations but did not differ for *RET*- versus non-*RET* inversions (table S4). Fusion^2B<20bp^-driven PTC accounted for approximately half (*n* = 34 of 66, 51.5%) of PTCs among individuals with the highest radiation doses [≥200 milligrays (mGy)] but only 9.8% (*n* = 15 of 153) among those with 1 to 99 mGy and 4.3% (*n* = 3 of 70) among unexposed individuals (Fig. 2B and table S4). In contrast, Fusion^≥3B≥1000bp^-driven PTC, *BRAF^V600E^*, and *RAS* mutations were less common with increasing radiation dose.

### Radiation dose and DNA DSB occurrence

**Fig. 3.**
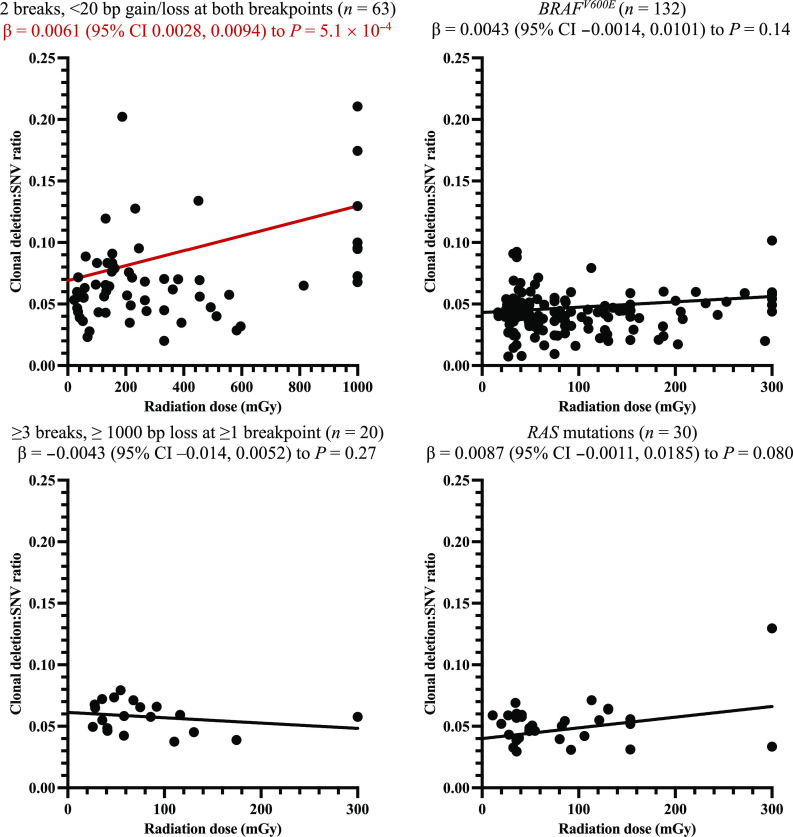
Relationship between radiation dose to the thyroid and DNA DSBs as measured by the clonal deletion:SNV ratio, by pattern of DNA damage that generated the PTC driver. Analyses excluded unexposed individuals (i.e., born >9 months after the accident). βs per 100 mGy [95% confidence interval (CI)] were estimated from linear regression models, with adjustment for sex and age at PTC diagnosis. Radiation dose outliers were truncated at 1000 mGy for PTC with fusion drivers with two breaks, <20 bp gain/loss at both breakpoints and 300 mGy for PTC with mutation drivers; deletion:SNV ratio outliers were truncated at 0.3. Note that *P* = 0.039 if dose was also truncated at 300 mGy for PTC with fusion drivers with two breaks, <20 bp gain/loss at both breakpoints. Red font and line indicate regression model with statistically significant parameter estimate (*P* < 0.05).

Because ionizing radiation exposure has consistently been found to increase the occurrence of small deletions, we hypothesized that radiation dose would be positively associated with the clonal deletion:SNV ratio only for radiation-induced tumors. Among exposed individuals, linear regression models adjusted for sex and age at PTC demonstrated a positive association between radiation dose and the clonal deletion:SNV ratio for Fusion^2B<20bp^-driven PTC [deletion:SNV ratio per 100 mGy, β (95% confidence interval): 0.0061 (0.0028 to 0.0094), *P* = 5.1 × 10^−4^] but no association for *BRAF^V600E^* [0.0043 (−0.0014 to 0.0101), *P* = 0.14] (Fig. 3). In exploratory analyses, the estimate was higher in *RET*- [β = 0.013 (0.0025 to 0.024), *P* = 0.017] than non-*RET* [β = 0.0042 (−0.00070 to 0.0090), *P* = 0.086] inversions or translocations [β = 0.0032 (0.00015 to 0.0063), *P* = 0.039] (table S5), whereas no statistically significant association was observed between radiation dose and the clonal deletion:SNV ratio for Fusion^≥3B≥1000bp^-driven PTC or *RAS* mutations.

### Timing of tumor initiation

**Fig. 4.**
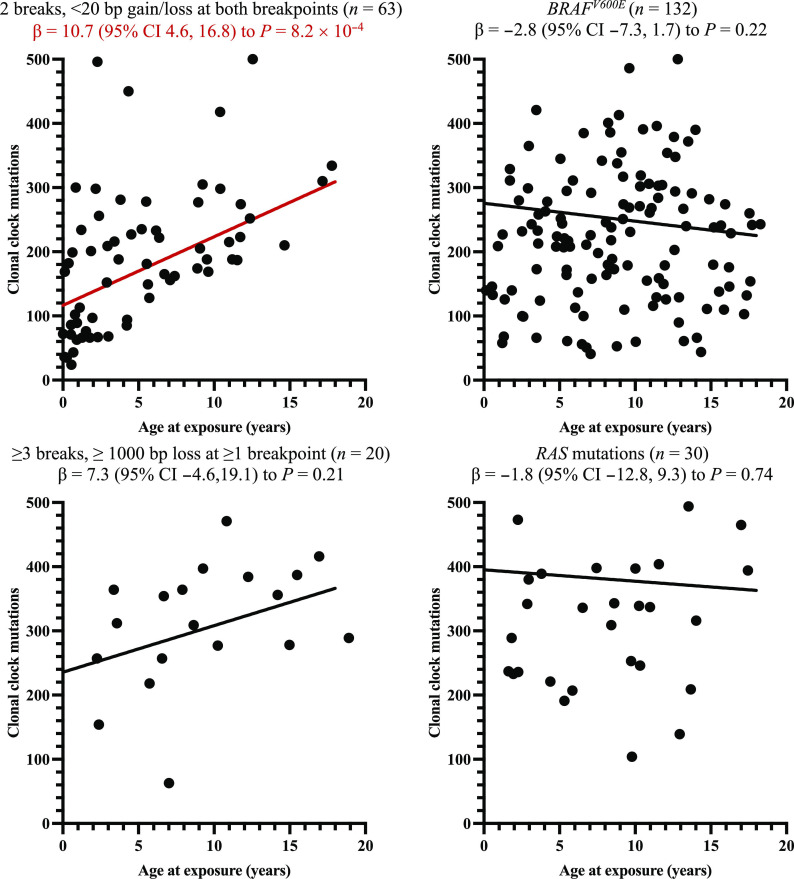
Relationship between age at the time of the Chornobyl accident and clonal clock mutations, by pattern of DNA damage that generated the PTC driver. Analyses excluded unexposed individuals (i.e., born >9 months after the accident). βs per year of age (95% CI) were estimated from linear regression models adjusted for sex and age at PTC. *P* values were calculated using likelihood ratio tests. Red font and line indicate regression model with statistically significant parameter estimate (*P* < 0.05).

Clock SBS mutational signatures (SBS1 and SBS5) represent mutations that accumulate with age (*10*–*12*); clonal clock mutations can therefore be analyzed to determine the age at which tumor initiation likely occurred (Fig. 1D). We hypothesized that radiation-induced tumors would be initiated at the time of the Chornobyl accident; thus, radiation-induced tumors would exhibit a positive relationship between clonal clock mutations and age at the time of the Chornobyl accident. Among exposed individuals, linear regression models adjusted for sex and age at PTC demonstrated a positive association between age at the time of the accident and clonal clock mutations for Fusion^2B<20bp^-driven PTC [clonal clock mutations SBS1 + SBS5 per year of age, β (95% confidence interval): 10.7 (4.6 to 16.8), *P* = 8.2 × 10^−4^] but no association for *BRAF^V600E^* [−2.8 (−7.3 to 1.7), *P* = 0.22] (Fig. 4 and table S6). In exploratory analyses, the association between age at the time of the accident and clonal clock mutations for Fusion^2B<20bp^-driven PTC was statistically significant for both *RET*- and non-*RET* inversions. In contrast, no statistically significant association was observed for translocations, *RAS* mutations, or Fusion^≥3B≥1000bp^-driven PTC. Sensitivity analyses separating SBS1 and SBS5 demonstrated consistent results for both signatures (table S6).

### Patient characteristics

**Fig. 5.**
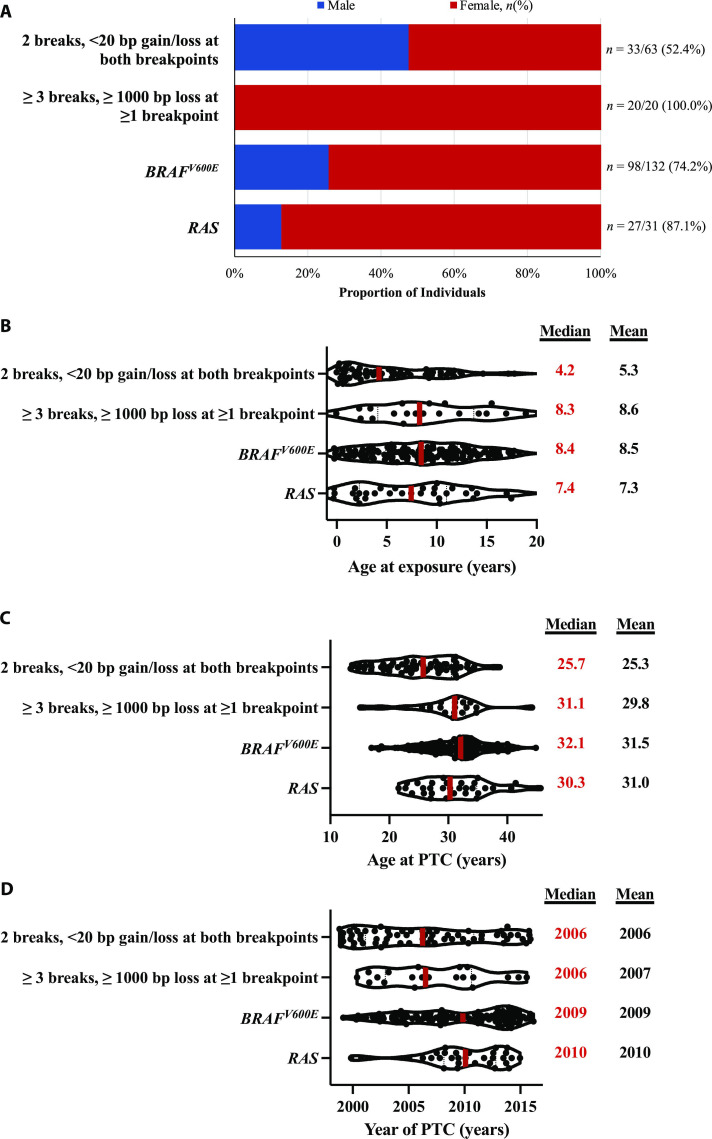
Patient characteristics among exposed individuals, by pattern of DNA damage that generated the PTC driver. Sex (**A**), age at exposure (**B**), age at PTC (**C**), and calendar year of PTC diagnosis (**D**).

Fusion^2B<20bp^-driven PTC occurred approximately evenly in males and females (52.4% female), whereas a notable female predominance, as is expected based on epidemiologic studies of sporadic PTC (*20*), was observed for other driver categories, including *BRAF^V600E^* (74.2% female), fusion_≥3B≥1000bp_ (100.0% female), and *RAS* mutations (87.1% female) (*P*_heterogeneity_ among main driver groups = 1.2 × 10^−5^) (Fig. 5A). Fusion^2B<20bp^-driven PTC tended to be younger at exposure (*P*_heterogeneity_ = 0.012) and at PTC diagnosis (*P*_heterogeneity_ = 1.3 × 10^−7^) and were diagnosed during an earlier calendar year (*P*_heterogeneity_ = 1.2 × 10^−4^) (Fig. 5, B to D), particularly for inversions (fig. S2, A to D). By comparison in TCGA, a similar female predominance (*P*_heterogeneity_ = 0.18) and comparable age at PTC diagnosis (*P*_heterogeneity_ = 0.63) were observed among all fusion/SV driver categories (fig. S2, E and F).

### *CLIP2* expression

Expression of *CLIP2* has been reported by others as a putative tumor biomarker for prior radiation exposure (*21*, *22*), but we previously reported no association between radiation dose and *CLIP2* in our overall study population (*4*). This lack of association persisted in the present study stratified by driver category (table S5).

## DISCUSSION

We used three orthogonal analyses—radiation dose distribution, relationship of radiation dose to occurrence of clonal small deletions, and timing of tumor initiation—to identify radiation-induced tumors after the Chornobyl nuclear power plant accident. Our results demonstrate that Fusion^2B<20bp^-driven PTCs as a group are consistent with having been caused by radiation, regardless of the specific gene fusions (i.e., involving *RET* versus other thyroid oncogenes). In contrast, we found no consistent evidence linking the group of PTCs driven by *BRAF^V600E^* mutations with radiation. Exploratory analyses, although based on smaller sample sizes, also suggested no consistent evidence that the group of PTCs driven by Fusion^≥3B≥1000bp^ or *RAS* mutations were caused by radiation. Our results are further supported by the observed sex ratios: Fusion^2B<20bp^-driven PTCs were distributed approximately evenly in males and females, which would be expected for an exposure such as ionizing radiation that is not sex-specific, whereas the PTCs with *BRAF^V600E^* mutations, Fusion^≥3B≥1000bp^, or *RAS* mutations occurred predominantly in females, which is consistent with the female predominance observed in the general population (*20*). The findings in this study have important implications, from providing insights into radiation-related tumorigenesis to helping to identify the true health impacts of radiation exposure in various settings, including nuclear accidents and medical and occupational exposures.

Initial studies of PTC in the decade following the Chornobyl accident reported an increased frequency of *RET-PTC3* (*NCOA4-RET*) compared with other *RET* fusions (*18*, *23*–*26*). Our study results explain the basis of these and other reports (*4*, *18*, *27*, *28*) on fusion/SV-driven PTC after the Chornobyl accident by demonstrating the importance of characterizing the pattern of DNA damage that generated the fusion/SV, rather than the specific genes involved. Specifically, we showed that most *RET-PTC3* were Fusion^2B<20bp^, whereas about 40% of *RET-PTC1* (*CCDC6-RET*) were Fusion^≥3B≥1000bp^, accounting for the excess of *RET-PTC3* in the early reports after the accident. Our study also shows that the radiation association is not restricted to *RET* fusions, because our findings were consistent for fusions, in particular inversions, involving other thyroid oncogenes, such as *BRAF* and *NTRK1*.

The association between radiation and Fusion^2B<20bp^-driven PTC is consistent with radiobiological data, suggesting that otherwise healthy cells exposed to ionizing radiation in this dose range (<1 Gy) should engage efficient DNA DSB repair without any delays or loss of major DNA fragments (*3*, *8*, *9*). Our exploratory analyses also supported a stronger radiation association for inversions rather than translocations, perhaps because ionizing radiation tracks are more likely to generate two DNA DSBs within the same chromosome versus on two separate chromosomes. Nevertheless, we have not yet identified a precise biomarker for radiation-induced tumors, because some Fusion^2B<20bp^-driven PTC occurred in unexposed individuals from both Chornobyl and TCGA, suggesting that this group of tumors is strongly enriched for radiation-induced PTC but still contains a small fraction of sporadic PTC caused by other factors. Fusion^≥3B≥1000bp^-driven PTCs likely represent long delays in repair and/or DNA fragment loss, which is more likely to occur when a cell is under substantial stress (i.e., not otherwise healthy or experiencing another type of exogenous or endogenous exposure that results in more widespread damage). However, we could not determine whether the number of DNA DSBs, the amount of gain/loss at the breakpoint, or both is the key characteristic for distinguishing radiation-induced tumors because we had small numbers of fusion/SV-driven PTCs generated from two DNA DSBs with ≥20 bp of gain/loss at the breakpoint or ≥3 DNA DSBs with <20 bp of gain/loss at the breakpoint. Unexposed individuals from both Chornobyl and TCGA had a higher proportion of fusion/SV-driven PTCs generated from two DNA DSBs with ≥20 bp of gain/loss, suggesting that the amount of gain/loss at the breakpoint might be a key feature. Larger sample sizes will be needed to understand the relative importance of the number of DNA DSBs versus the amount of gain/loss at the breakpoint to enable identification of radiation-induced tumors.

Our current data do not support a substantial causal role for radiation-induced damage in the generation of PTCs with *BRAF^V600E^* or *RAS* mutations, a finding consistent with the lack of excess SNVs in previous reports of both normal (*3*) and tumor (*4*–*6*) tissues following ionizing radiation exposure. Nevertheless, because we have not yet identified a unique biomarker for radiation-induced tumors, we cannot rule out the possibility that radioactive fallout from the Chornobyl accident caused a small fraction of these PTCs.

This study includes a large sample size, detailed data on radiation exposure to the thyroid from radioactive iodine (^131^I)–contaminated fallout across a range of exposure levels, inclusion of unexposed individuals (born >9 months after the accident) and TCGA data as a reference group, consistent histologic confirmation of the tumors through the Chornobyl Tissue Bank (*29*, *30*), and WGS data. Despite the strength and consistency of our findings, they should be interpreted with caution. The low mutational burden of PTC and relatively low radiation doses for most individuals in our study resulted in low numbers of radiation-associated mutations, thus limiting our statistical power, and we did not have sufficient sample size for more detailed regression modeling of the deletions by deletion size, insertion-deletion signature 8 [ID8, thought to be related to nonhomologous end-joining (NHEJ) repair of DNA DSBs] or ID6 (thought to be related to alternative end joining of DSBs), or to consider SBS18 (related to reactive oxygen species generation), nondriver SVs, or somatic copy number alterations (SCNAs), including evaluation of the fraction of genome altered (*10*, *11*). Because the Chornobyl Tissue Bank did not begin collecting specimens until over a decade after the accident, we cannot address the true latency of radiation-induced PTC (*29*, *30*). Some dose estimates may be misclassified because a large fraction of individuals in the study were not directly interviewed regarding their location and dietary habits in the months immediately following the accident. The age-specific patterns we observed raise the potential of a combined effect of young age at radiation exposure and vulnerability of the thyroid to damage during development, but these analyses are complicated by the known decrease in the proportion of fusion-driven PTCs with increasing age at diagnosis in the general population, particularly since PTCs from TCGA were older on average than Chornobyl (*4*, *7*, *31*). Last, PTCs typically have only a single oncogenic driver (*4*, *7*), and it is not clear whether our findings would apply to other tissue types that more commonly exhibit multiple drivers (*32*). Future efforts that include individuals with higher radiation doses, a larger number of age-matched individuals who are unexposed, and other tumor types are essential for understanding the generalizability of our findings.

In conclusion, our study findings support a potential causal role for ionizing radiation in the occurrence of a large fraction of the Fusion^2B<20bp^-driven PTCs after the Chornobyl accident, but rarely, if at all, *BRAF^V600E^* mutations, *RAS* mutations, or Fusion^≥3B≥1000bp^-driven PTC. With extension to other radiation exposures and doses as well as tumor types, these results hold promise for understanding the true burden of cancer following ionizing radiation from occupational, medical, and environmental exposures.

## MATERIALS AND METHODS

### Study population

The primary study population for this analysis included *n* = 355 individuals with high-quality WGS data (*4*) from a fresh frozen, pretreatment, histopathologically confirmed PTC sample collected by the Chornobyl Tissue Bank (*29*, *30*). Eligibility for sample collection included residence in one of the most contaminated territories (oblasts or states) of Ukraine, specifically, Zhytomyr, Kyiv, or Chernihiv; in utero or <19 years of age on 26 April 1986 (cases) or born >9 months after the accident (comparison population of unexposed individuals); histopathologically confirmed diagnosis of a first primary PTC based on review of tumor tissue by an international panel of experts; and medical record data documenting no prior cancer history, availability of ^131^I dose estimates, and availability of nontumor thyroid tissue and/or a blood sample collected at the time of PTC diagnosis.

^131^I doses to the thyroid were reconstructed by an international team of dosimetry experts based on methods described previously for this and other studies of radiation-related health risks following the accident (*4*, *33*–*36*). Dose estimation varied based on data availability but included individual thyroid radioactivity measurements taken in May and June 1986, personal interviews regarding residential history and intake of milk and green leafy vegetables, and results of radio-ecological modeling; individual thyroid radioactivity measurements (without personal interview); thyroid radioactivity measurements on different individuals who lived in the same residential area; or maternal exposure data for individuals who were in utero at the time of the accident.

Participants (or guardians for minors) provided informed consent for donation and broad research use of their materials through the Chornobyl Tissue Bank, and this ongoing study was approved by Institutional Review Boards at the tissue collection center (V.P. Komisarenko Institute of Endocrinology and Metabolism of the National Academy of Medical Sciences of Ukraine in Kyiv, Ukraine; protocol no. IRB00003633) and an Ethical Review Panel at the United States National Cancer Institute (protocol no. 22G019-15).

We also included a secondary study population derived from previously published data from TCGA, the only other published large-scale study of PTCs with driver characterization and WGS data (*7*).

### Molecular data

For the Chornobyl study, simple somatic variants (SNVs and small indels), SVs, SigProfiler mutational signatures, and PTC drivers were ascertained from previously published supplemental data (*4*). Cancer cell fraction ≥0.9 was used to define clonality for the clonal deletion:SNV ratio and clonal clock mutational signatures (*4*).

The prior PTC driver identification was extended by analyzing the WGS data [available in the Genomic Data Commons accessed through the database of Genotypes and Phenotypes (dbGaP), accession phs001134; www.ncbi.nlm.nih.gov/gap/] and somatic variant calls (available in the previously published supplemental data) (*4*) to characterize each tumor (*n* = 355) by the pattern of DNA damage that generated the driver. Fusion/SV drivers (*n* = 140) were further classified according to various features of the repair using the previously generated clustering of the SV calls into SV events (*4*). Clustered SV events were grouped if they involved the same chromosomes. All SV breakpoints were manually visualized in the Integrative Genomics Viewer (IGV; https://igv.org/), and clustered SV events were confirmed by manual recreation of the final repaired chromosome(s). Very few special repair scenarios were identified (Table 1), including large deletions (≥1000 bp), large insertions (≥1000 bp), tandem duplications (≥1000 bp), templated insertions, dicentric chromosome fragments, and chromothripsis. The remaining fusion driver events were then classified according to the number of DNA DSBs and the amount of gain or loss at the breakpoints. Rarely, individual SV calls went unidentified within the clustered SV events because the missing SV call was not detected by the SV callers and not found by manual inspection in IGV (3 of the 140 fusion events had at least one missing SV call), which is likely due to the SV call occurring in repeat/low complexity regions of the genome. Classification of the three clustered SV/fusion driver events was unaffected since all three already had been identified as having ≥3 DNA DSBs with ≥1000 bp loss at ≥1 breakpoint.

Mutation drivers were classified as SBSs, dinucleotide substitutions, small insertions, small deletions, and multiple mutations (Table 1). The multiple mutations occur in tumor suppressor genes (*APC*, *DICER1*, and *TSC2*), which require biallelic loss, and included combinations of SBS + small deletion (*n* = 3), small deletions + SCNA (*n* = 1), SBS + SCNA (*n* = 1), and germline mutation + SCNA (*n* = 1).

The TCGA fusion/SV driven tumors with high-quality WGS data available and without known radiation exposure (*n* = 69) were also further characterized to determine the pattern of DNA damage that generated the driver. WGS BAM files were downloaded from the Genomic Data Commons (accessed through dbGaP, accession phs000178). When multiple BAM files were available for a given sample, the BAM with the high depth was used. MANTA 1.6.0 was run in tumor-normal mode, within a Singularity version CE 4.1.5 container. Data processing, formatting, and preparation were performed via vArmyKnife version 3.3.147. As was done in the Chornobyl dataset, the SV calls were grouped into SV events. All SV breakpoints were manually visualized in IGV to confirm all SV calls within each clustered SV event and then the fusion was classified according to the same rules used for the Chornobyl dataset. Five of the 69 clustered SV events had at least one missing SV call, which did not affect the fusion classification since all five already had been identified as having ≥3 DNA DSBs with ≥1000 bp loss at ≥1 breakpoint.

### Statistical analysis

Descriptive statistics were used to summarize distributions of patient, tumor, and molecular characteristics. Because of small samples sizes for some categories, Fisher’s exact tests were used to compare the distributions of fusion/SV drivers by the pattern of DNA damage that generated them between exposed individuals and unexposed individuals from Chornobyl and TCGA. Multivariable linear regression models were used to investigate the relationship between age at the time of the Chornobyl accident and clonal clock mutations and to investigate the relationship between radiation dose and clonal deletion:SNV ratio, whereas polytomous logistic regression models were used to test for heterogeneity among the main driver groups according to selected characteristics. All models were adjusted for age at PTC (continuous) and sex. Two-sided *P* values were generated using likelihood ratio tests, comparing model fit with and without the variable of interest. Analyses were conducted using SAS version 9.4 (Cary, NC).

## References

[1] International Agency for Research on Cancer. (Lyon, France, 2012).

[2] United Nations Scientific Committee on the Effects of Atomic Radiation (UNSCEAR), "Effects of Ionizing Radiation: United Nations Scientific Committee on the Effects of Atomic Radiation: UNSCEAR 2006 Report, Volume 1—Report to the General Assembly, with Scientific Annexes A and B" (United Nations Publication, 2008).

[3] J. Youk, H. W. Kwon, J. Lim, E. Kim, T. Kim, R. Kim, S. Park, K. Yi, C. H. Nam, S. Jeon, Y. An, J. Choi, H. Na, E. S. Lee, Y. Cho, D. W. Min, H. Kim, Y. R. Kang, S. H. Choi, M. J. Bae, C. G. Lee, J. G. Kim, Y. S. Kim, T. Yu, W. C. Lee, J. Y. Shin, D. S. Lee, T. Y. Kim, T. Ku, S. Y. Kim, J. H. Lee, B. K. Koo, H. Lee, O. V. Yi, E. C. Han, J. H. Chang, K. S. Kim, T. G. Son, Y. S. Ju, Quantitative and qualitative mutational impact of ionizing radiation on normal cells. Cell Genom. 4, 100499 (2024). 38359788 10.1016/j.xgen.2024.100499 PMC10879144

[4] L. M. Morton, D. M. Karyadi, C. Stewart, T. I. Bogdanova, E. T. Dawson, M. K. Steinberg, J. Dai, S. W. Hartley, S. J. Schonfeld, J. N. Sampson, Y. E. Maruvka, V. Kapoor, D. A. Ramsden, J. Carvajal-Garcia, C. M. Perou, J. S. Parker, M. Krznaric, M. Yeager, J. F. Boland, A. Hutchinson, B. D. Hicks, C. L. Dagnall, J. M. Gastier-Foster, J. Bowen, O. Lee, M. J. Machiela, E. K. Cahoon, A. V. Brenner, K. Mabuchi, V. Drozdovitch, S. Masiuk, M. Chepurny, L. Y. Zurnadzhy, M. Hatch, A. Berrington de Gonzalez, G. A. Thomas, M. D. Tronko, G. Getz, S. J. Chanock, Radiation-related genomic profile of papillary thyroid carcinoma after the Chernobyl accident. Science 372, eabg2538 (2021). 33888599 10.1126/science.abg2538 PMC9022889

[5] S. Behjati, G. Gundem, D. C. Wedge, N. D. Roberts, P. S. Tarpey, S. L. Cooke, P. Van Loo, L. B. Alexandrov, M. Ramakrishna, H. Davies, S. Nik-Zainal, C. Hardy, C. Latimer, K. M. Raine, L. Stebbings, A. Menzies, D. Jones, R. Shepherd, A. P. Butler, J. W. Teague, M. Jorgensen, B. Khatri, N. Pillay, A. Shlien, P. A. Futreal, C. Badie, ICGC Prostate Group, U. McDermott, G. S. Bova, A. L. Richardson, A. M. Flanagan, M. R. Stratton, P. J. Campbell, Mutational signatures of ionizing radiation in second malignancies. Nat. Commun. 7, 12605 (2016). 27615322 10.1038/ncomms12605 PMC5027243

[6] E. Kocakavuk, K. J. Anderson, F. S. Varn, K. C. Johnson, S. B. Amin, E. P. Sulman, M. P. Lolkema, F. P. Barthel, R. G. W. Verhaak, Radiotherapy is associated with a deletion signature that contributes to poor outcomes in patients with cancer. Nat. Genet. 53, 1088–1096 (2021). 34045764 10.1038/s41588-021-00874-3 PMC8483261

[7] The Cancer Genome Atlas Research Network, Integrated genomic characterization of papillary thyroid carcinoma. Cell 159, 676–690 (2014). 25417114 10.1016/j.cell.2014.09.050 PMC4243044

[8] J. A. Hussmann, J. Ling, P. Ravisankar, J. Yan, A. Cirincione, A. Xu, D. Simpson, D. Yang, A. Bothmer, C. Cotta-Ramusino, J. S. Weissman, B. Adamson, Mapping the genetic landscape of DNA double-strand break repair. Cell 184, 5653–5669.e25 (2021). 34672952 10.1016/j.cell.2021.10.002 PMC9074467

[9] W. Feng, D. A. Simpson, J. E. Cho, J. Carvajal-Garcia, C. M. Smith, K. M. Headley, N. Hathaway, D. A. Ramsden, G. P. Gupta, Marker-free quantification of repair pathway utilization at Cas9-induced double-strand breaks. Nucleic Acids Res. 49, 5095–5105 (2021). 33963863 10.1093/nar/gkab299 PMC8136827

[10] L. B. Alexandrov, S. Nik-Zainal, D. C. Wedge, P. J. Campbell, M. R. Stratton, Deciphering signatures of mutational processes operative in human cancer. Cell Rep. 3, 246–259 (2013). 23318258 10.1016/j.celrep.2012.12.008 PMC3588146

[11] L. B. Alexandrov, J. Kim, N. J. Haradhvala, M. N. Huang, A. W. Tian Ng, Y. Wu, A. Boot, K. R. Covington, D. A. Gordenin, E. N. Bergstrom, S. M. A. Islam, N. Lopez-Bigas, L. J. Klimczak, J. R. McPherson, S. Morganella, R. Sabarinathan, D. A. Wheeler, V. Mustonen, PCAWG Mutational Signatures Working Group, G. Getz, S. G. Rozen, M. R. Stratton, PCAWG Consortium, The repertoire of mutational signatures in human cancer. Nature 578, 94–101 (2020). 32025018 10.1038/s41586-020-1943-3 PMC7054213

[12] N. Spisak, M. de Manuel, W. Milligan, G. Sella, M. Przeworski, The clock-like accumulation of germline and somatic mutations can arise from the interplay of DNA damage and repair. PLOS Biol. 22, e3002678 (2024). 38885262 10.1371/journal.pbio.3002678 PMC11213356

[13] E. Ostroumova, J. Schuz, A. Kesminiene, Future of Chernobyl research: The urgency for consolidated action. Lancet 395, 1037–1038 (2020). 10.1016/S0140-6736(20)30675-9 PMC7270813 32199076

[14] T. Nakaya, K. Takahashi, H. Takahashi, S. Yasumura, T. Ohira, H. Shimura, S. Suzuki, S. Suzuki, M. Iwadate, S. Yokoya, H. Ohto, K. Kamiya, Revisiting the geographical distribution of thyroid cancer incidence in Fukushima prefecture: Analysis of data from the second- and third-round thyroid ultrasound examination. J. Epidemiol. 32, S76–S83 (2022). 36464303 10.2188/jea.JE20210165 PMC9703926

[15] T. I. Bogdanova, V. A. Saenko, Y. Hashimoto, M. Hirokawa, L. Y. Zurnadzhy, T. Hayashi, M. Ito, M. Iwadate, N. Mitsutake, T. I. Rogounovitch, A. Sakamoto, H. Naganuma, A. Miyauchi, M. D. Tronko, G. Thomas, S. Yamashita, S. Suzuki, Papillary thyroid carcinoma in Ukraine after Chernobyl and in Japan after Fukushima: Different histopathological scenarios. Thyroid 31, 1322–1334 (2021). 33143557 10.1089/thy.2020.0308

[16] B. D. Breitenstein, The probability that a specific cancer and a specified radiation exposure are causally related. Health Phys. 55, 397–398 (1988). 3410710 10.1097/00004032-198808000-00038

[17] S. Niu, P. Deboodt, H. Zeeb, Eds., *Approaches to attribution of detrimental health effects to occupational ionizing radiation exposure and their application in compensation programmes for cancer: A practical guide*, (Jointly prepared by the International Atomic Energy Agency, the International Labour Organization and the World Health Organization, Geneva, 2010), vol. Occupational Safety and Health Series, No. 73.

[18] T. I. Rogounovitch, S. V. Mankovskaya, M. V. Fridman, T. A. Leonova, V. A. Kondratovitch, N. E. Konoplya, S. Yamashita, N. Mitsutake, V. A. Saenko, Major oncogenic drivers and their clinicopathological correlations in sporadic childhood papillary thyroid carcinoma in Belarus. Cancers 13, 3374 (2021). 34282777 10.3390/cancers13133374 PMC8268670

[19] J. H. Lubin, M. J. Adams, R. Shore, E. Holmberg, A. B. Schneider, M. M. Hawkins, L. L. Robison, P. D. Inskip, M. Lundell, R. Johansson, R. A. Kleinerman, F. de Vathaire, L. Damber, S. Sadetzki, M. Tucker, R. Sakata, L. H. S. Veiga, Thyroid cancer following childhood low-dose radiation exposure: A pooled analysis of nine cohorts. J. Clin. Endocrinol. Metab. 102, 2575–2583 (2017). 28323979 10.1210/jc.2016-3529 PMC5505197

[20] H. Lim, S. S. Devesa, J. A. Sosa, D. Check, C. M. Kitahara, Trends in thyroid cancer incidence and mortality in the United States, 1974-2013. JAMA 317, 1338–1348 (2017). 28362912 10.1001/jama.2017.2719 PMC8216772

[21] M. Selmansberger, A. Feuchtinger, L. Zurnadzhy, A. Michna, J. C. Kaiser, M. Abend, A. Brenner, T. Bogdanova, A. Walch, K. Unger, H. Zitzelsberger, J. Hess, CLIP2 as radiation biomarker in papillary thyroid carcinoma. Oncogene 34, 3917–3925 (2015). 25284583 10.1038/onc.2014.311

[22] M. Selmansberger, J. C. Kaiser, J. Hess, D. Guthlin, I. Likhtarev, V. Shpak, M. Tronko, A. Brenner, M. Abend, M. Blettner, K. Unger, P. Jacob, H. Zitzelsberger, Dose-dependent expression of CLIP2 in post-Chernobyl papillary thyroid carcinomas. Carcinogenesis 36, 748–756 (2015). 25957251 10.1093/carcin/bgv043 PMC4496450

[23] E. D. Williams, A. Abrosimov, T. Bogdanova, E. P. Demidchik, M. Ito, V. LiVolsi, E. Lushnikov, J. Rosai, Y. Sidorov, M. D. Tronko, A. F. Tsyb, S. L. Vowler, G. A. Thomas, Thyroid carcinoma after Chernobyl latent period, morphology and aggressiveness. Br. J. Cancer 90, 2219–2224 (2004). 15150580 10.1038/sj.bjc.6601860 PMC2409486

[24] G. A. Thomas, H. Bunnell, H. A. Cook, E. D. Williams, A. Nerovnya, E. D. Cherstvoy, N. D. Tronko, T. I. Bogdanova, G. Chiappetta, G. Viglietto, F. Pentimalli, G. Salvatore, A. Fusco, M. Santoro, G. Vecchio, High prevalence of RET/PTC rearrangements in Ukrainian and Belarussian post-Chernobyl thyroid papillary carcinomas: A strong correlation between RET/PTC3 and the solid-follicular variant. J. Clin. Endocrinol. Metab. 84, 4232–4238 (1999). 10566678 10.1210/jcem.84.11.6129

[25] H. M. Rabes, E. P. Demidchik, J. D. Sidorow, E. Lengfelder, C. Beimfohr, D. Hoelzel, S. Klugbauer, Pattern of radiation-induced RET and NTRK1 rearrangements in 191 post-chernobyl papillary thyroid carcinomas: Biological, phenotypic, and clinical implications. Clin. Cancer Res. 6, 1093–1103 (2000). 10741739

[26] Y. E. Nikiforov, J. M. Rowland, K. E. Bove, H. Monforte-Munoz, J. A. Fagin, Distinct pattern of ret oncogene rearrangements in morphological variants of radiation-induced and sporadic thyroid papillary carcinomas in children. Cancer Res. 57, 1690–1694 (1997). 9135009

[27] A. A. Efanov, A. V. Brenner, T. I. Bogdanova, L. M. Kelly, P. Liu, M. P. Little, A. I. Wald, M. Hatch, L. Y. Zurnadzy, M. N. Nikiforova, V. Drozdovitch, R. Leeman-Neill, K. Mabuchi, M. D. Tronko, S. J. Chanock, Y. E. Nikiforov, Investigation of the relationship between radiation dose and gene mutations and fusions in post-Chernobyl thyroid cancer. J. Natl. Cancer Inst. 110, 371–378 (2018). 29165687 10.1093/jnci/djx209 PMC6059206

[28] J. C. Ricarte-Filho, S. Li, M. E. Garcia-Rendueles, C. Montero-Conde, F. Voza, J. A. Knauf, A. Heguy, A. Viale, T. Bogdanova, G. A. Thomas, C. E. Mason, J. A. Fagin, Identification of kinase fusion oncogenes in post-Chernobyl radiation-induced thyroid cancers. J. Clin. Invest. 123, 4935–4944 (2013). 24135138 10.1172/JCI69766 PMC3809792

[29] G. A. Thomas, E. D. Williams, Thyroid tumor banks. Science 289, 2283 (2000). 11041794 10.1126/science.289.5488.2283a

[30] G. A. Thomas, The Chernobyl Tissue Bank: Integrating research on radiation-induced thyroid cancer. J. Radiol. Prot. 32, N77–N80 (2012). 22394998 10.1088/0952-4746/32/1/N77

[31] A. T. Franco, J. C. Ricarte-Filho, A. Isaza, Z. Jones, N. Jain, S. Mostoufi-Moab, L. Surrey, T. W. Laetsch, M. M. Li, J. C. DeHart, E. Reichenberger, D. Taylor, K. Kazahaya, N. S. Adzick, A. J. Bauer, Fusion oncogenes are associated with increased metastatic capacity and persistent disease in pediatric thyroid cancers. J. Clin. Oncol. 40, 1081–1090 (2022). 35015563 10.1200/JCO.21.01861 PMC8966969

[32] E. Rheinbay, M. M. Nielsen, F. Abascal, J. A. Wala, O. Shapira, G. Tiao, H. Hornshoj, J. M. Hess, R. I. Juul, Z. Lin, L. Feuerbach, R. Sabarinathan, T. Madsen, J. Kim, L. Mularoni, S. Shuai, A. Lanzos, C. Herrmann, Y. E. Maruvka, C. Shen, S. B. Amin, P. Bandopadhayay, J. Bertl, K. A. Boroevich, J. Busanovich, J. Carlevaro-Fita, D. Chakravarty, C. W. Y. Chan, D. Craft, P. Dhingra, K. Diamanti, N. A. Fonseca, A. Gonzalez-Perez, Q. Guo, M. P. Hamilton, N. J. Haradhvala, C. Hong, K. Isaev, T. A. Johnson, M. Juul, A. Kahles, A. Kahraman, Y. Kim, J. Komorowski, K. Kumar, S. Kumar, D. Lee, K. V. Lehmann, Y. Li, E. M. Liu, L. Lochovsky, K. Park, O. Pich, N. D. Roberts, G. Saksena, S. E. Schumacher, N. Sidiropoulos, L. Sieverling, N. Sinnott-Armstrong, C. Stewart, D. Tamborero, J. M. C. Tubio, H. M. Umer, L. Uuskula-Reimand, C. Wadelius, L. Wadi, X. Yao, C. Z. Zhang, J. Zhang, J. E. Haber, A. Hobolth, M. Imielinski, M. Kellis, M. S. Lawrence, C. von Mering, H. Nakagawa, B. J. Raphael, M. A. Rubin, C. Sander, L. D. Stein, J. M. Stuart, T. Tsunoda, D. A. Wheeler, R. Johnson, J. Reimand, M. Gerstein, E. Khurana, P. J. Campbell, N. Lopez-Bigas, PCAWG Drivers and Functional Interpretation Working Group, PCAWG Structural Variation Working Group, J. Weischenfeldt, R. Beroukhim, I. Martincorena, J. S. Pedersen, G. Getz, PCAWG Consortium, Analyses of non-coding somatic drivers in 2,658 cancer whole genomes. Nature 578, 102–111 (2020).32025015

[33] I. Likhtarov, G. Thomas, L. Kovgan, S. Masiuk, M. Chepurny, O. Ivanova, V. Gerasymenko, M. Tronko, T. Bogdanova, A. Bouville, Reconstruction of individual thyroid doses to the Ukrainian subjects enrolled in the Chernobyl Tissue Bank. Radiat. Prot. Dosimetry 156, 407–423 (2013). 23595409 10.1093/rpd/nct096

[34] S. Masiuk, M. Chepurny, V. Buderatska, O. Ivanova, Z. Boiko, N. Zhadan, M. Hatch, E. K. Cahoon, G. Zamotayeva, V. Shpak, M. Tronko, V. Drozdovitch, Assessment of internal exposure to 131I and short-lived radioiodine isotopes and associated uncertainties in the Ukrainian cohort of persons exposed in utero. J. Radiat. Res. 63, 364–377 (2022). 35301522 10.1093/jrr/rrac007 PMC9124623

[35] S. Masiuk, M. Chepurny, V. Buderatska, O. Ivanova, Z. Boiko, N. Zhadan, K. Mabuchi, E. K. Cahoon, M. P. Little, A. Kukush, T. Bogdanova, V. Shpak, G. Zamotayeva, M. Tronko, V. Drozdovitch, Exposure to the thyroid from intake of radioiodine isotopes after the chornobyl accident. Report I: Revised doses and associated uncertainties for the Ukrainian-American cohort. Radiat. Res. 199, 61–73 (2023). 36366807 10.1667/RADE-21-00152.1 PMC9899004

[36] S. Masiuk, M. Chepurny, V. Buderatska, O. Ivanova, Z. Boiko, N. Zhadan, H. Chornovol, M. Bolgov, V. Shpak, M. Tronko, E. K. Cahoon, S. J. Chanock, T. Bogdanova, L. M. Morton, V. Drozdovitch, Thyroid doses for the Chornobyl Tissue Bank: Improved estimates based on revised methodology and individual residence and diet history. Radiat. Environ. Biophys. 64, 85–98 (2025). 39699684 10.1007/s00411-024-01099-8 PMC11971128

