# Supplementary material for: Distinctive molecular features of radiation-induced thyroid cancers
Source: Sci Adv. 2025 Aug 22;11(34):eadw7680. doi: 10.1126/sciadv.adw7680 (PMC12372901; doi:10.1126/sciadv.adw7680)
Supplement: Supplementary file 1 — Figs. S1 and S2 Tables S1 to S6 Legend for data S1 [file sciadv.adw7680_sm.pdf]

Science Advances
AAAS

Supplementary Materials for

Distinctive molecular features of radiation-induced thyroid cancers

Danielle M. Karyadi *et al.*

Corresponding author: Lindsay M. Morton, mortonli@mail.nih.gov

Sci. Adv. **11**, eadw7680 (2025)
DOI: 10.1126/sciadv.adw7680

The PDF file includes:

Figs. S1 and S2
Tables S1 to S6
Legend for data S1

Other Supplementary Material for this manuscript includes the following:

Data S1

A

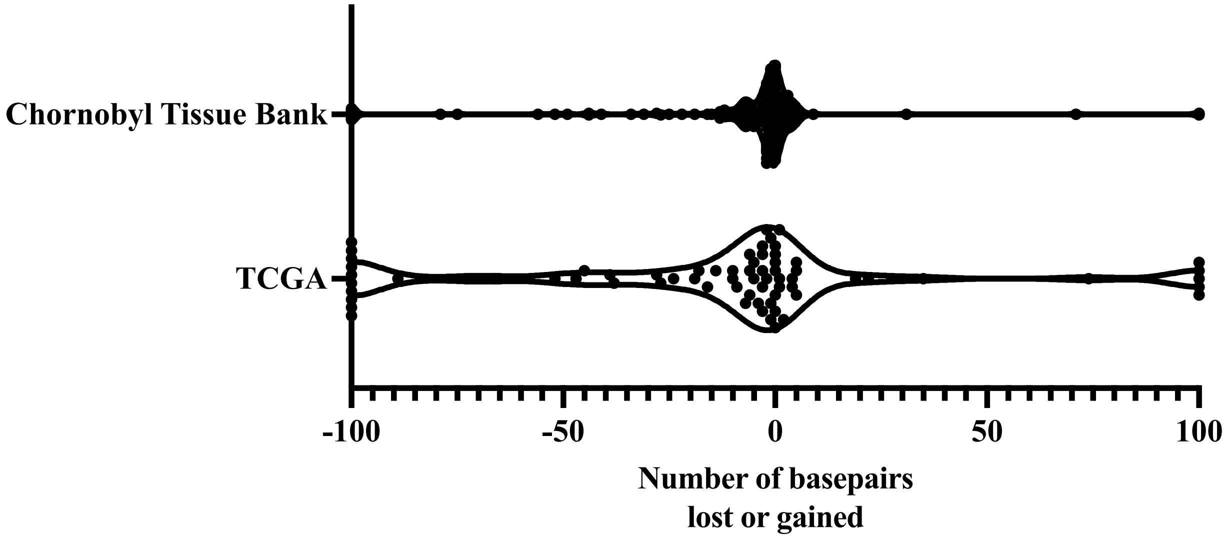

B

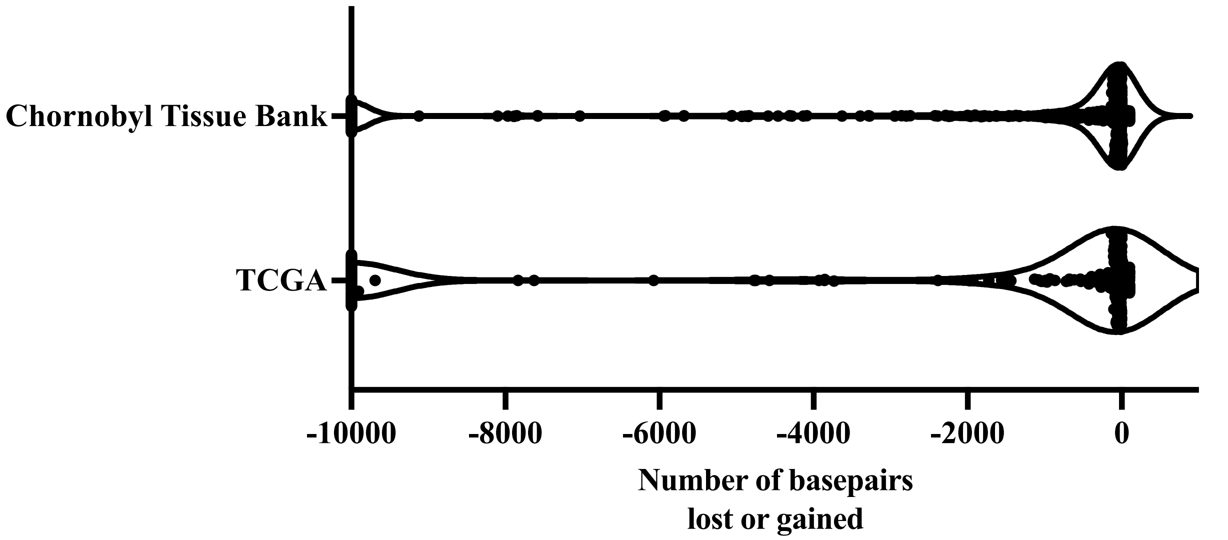

Supplementary Fig. 1. Distribution of the amount of gain or loss at the breakpoints for fusion/SV drivers in the Chornobyl Tissue Bank and TCGA. Fusion/SV drivers generated from 2 (A) versus ≥3 (B) DNA DSBs.

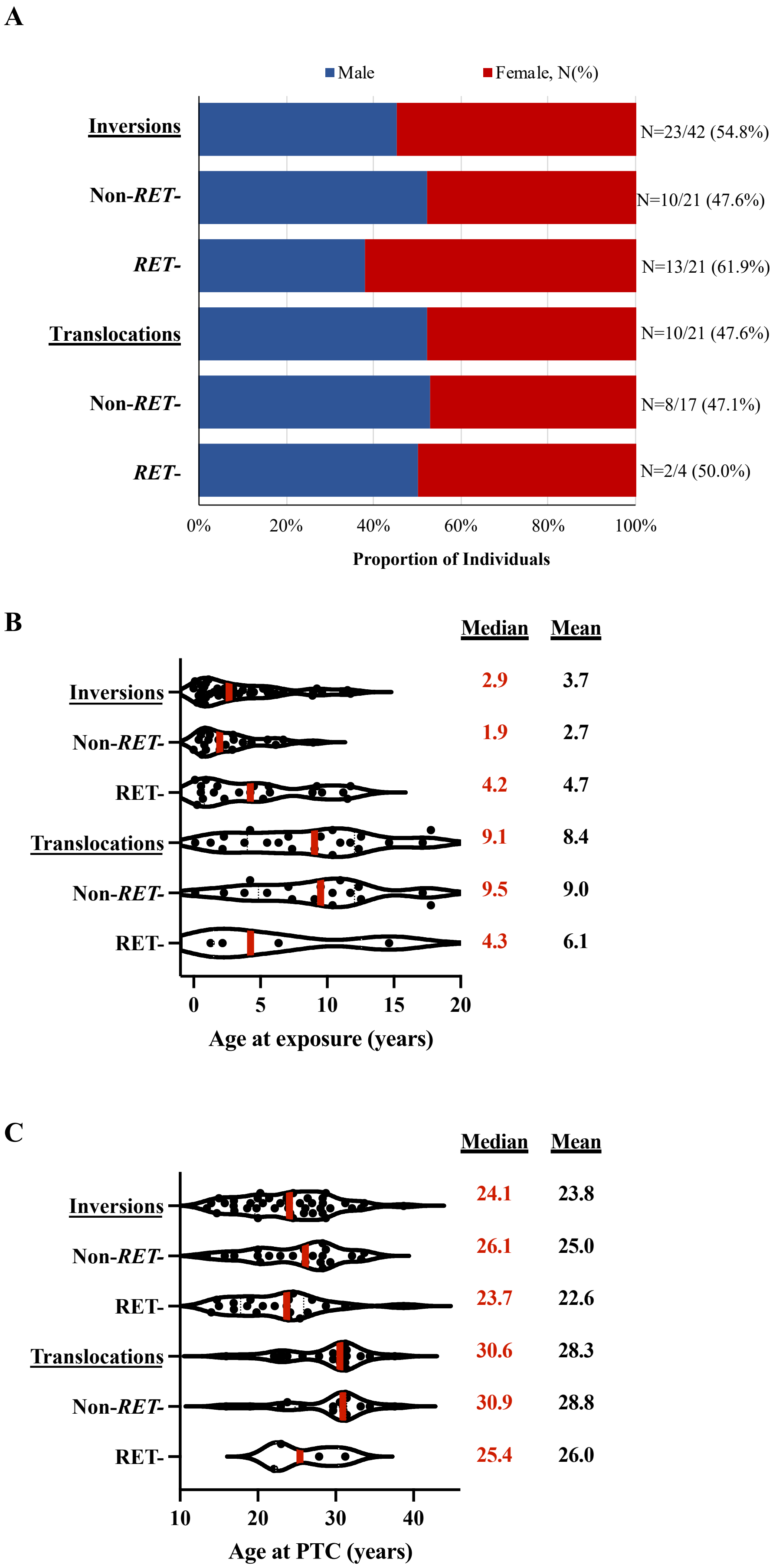
A
Male
Female, N(%)
Inversions
Non-RET-
RET-
Translocations
Non-RET-
RET-
N=23/42 (54.8%)
N=10/21 (47.6%)
N=13/21 (61.9%)
N=10/21 (47.6%)
N=8/17 (47.1%)
N=2/4 (50.0%)
0%
20%
40%
60%
80%
100%
Proportion of Individuals
B
Median
Mean
Inversions
Non-RET-
RET-
Translocations
Non-RET-
RET-
2.9
3.7
1.9
2.7
4.2
4.7
9.1
8.4
9.5
9.0
4.3
6.1
0
5
10
15
20
Age at exposure (years)
C
Median
Mean
Inversions
Non-RET-
RET-
Translocations
Non-RET-
RET-
24.1
23.8
26.1
25.0
23.7
22.6
30.6
28.3
30.9
28.8
25.4
26.0
10
20
30
40
Age at PTC (years)

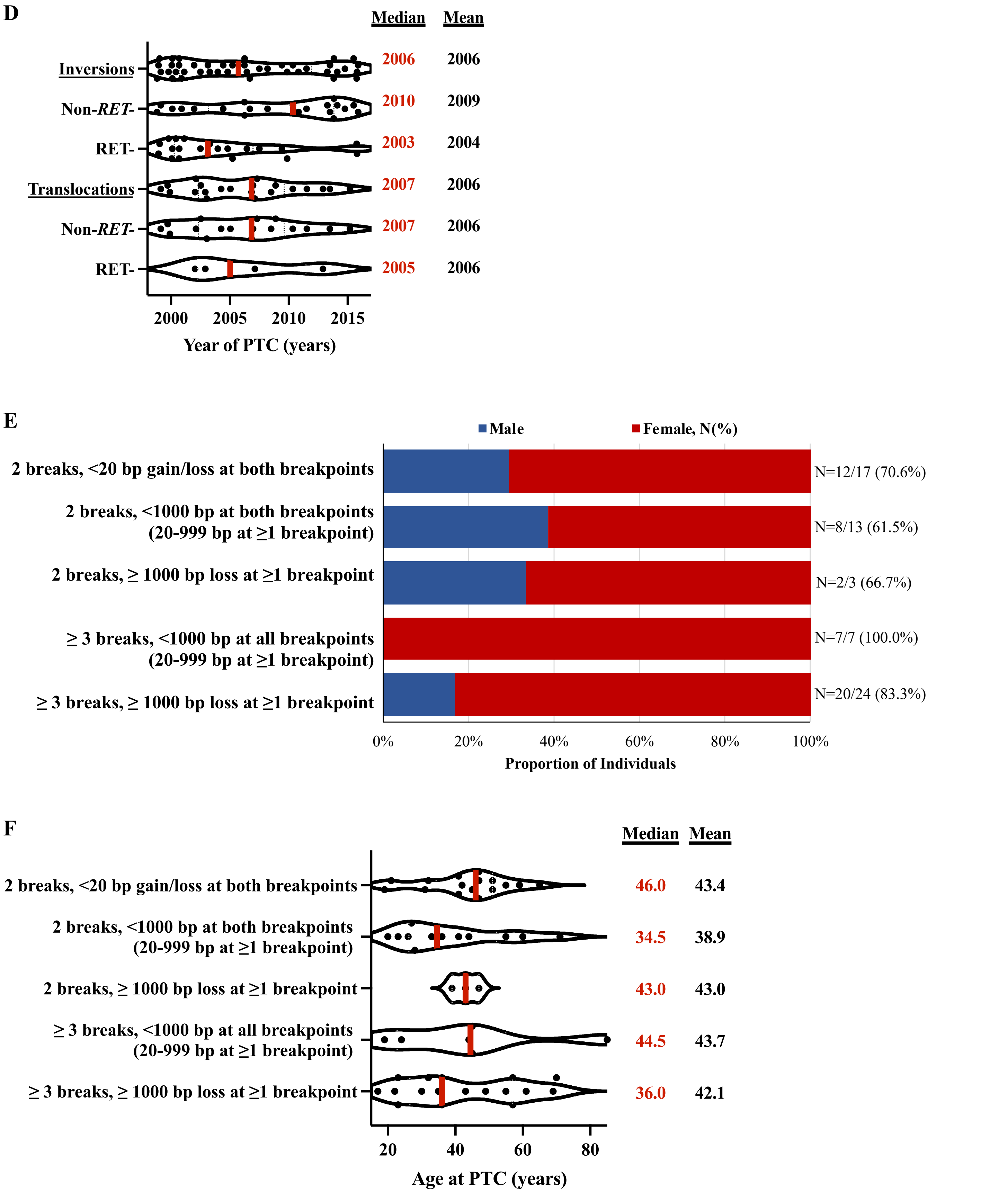

Supplementary Fig. 2. Patient characteristics for PTCs, by the pattern of DNA damage that generated the PTC driver. Sex (A), age at exposure (B), age at PTC (C), and calendar year of PTC diagnosis (D) among exposed individuals from the Chornobyl Tissue Bank with fusion/SV drivers generated from 2 breaks with <20 bp of gain/loss at both breakpoints, by inversion versus translocation and involvement of *RET*- versus other thyroid oncogenes. Sex (E) and age at PTC (F) in TCGA (categories with N>1 are presented).

Supplementary Table 1. Distribution of 355 PTCs from the Chornobyl Tissue Bank by the pattern of DNA damage that generated the driver, by driver gene and by fusion partner for fusion/SV drivers

Driver type<br>Detailed classification		N (%)*	N (%)*	N (%)*	N (%)*	N (%)*	N (%)*	N (%)*	N (%)*	N (%)*	N (%)*	N (%)*	N (%)*	N (%)*	N (%)*	N (%)*	N (%)*	N (%)*	N (%)*	N (%)*	N (%)*	N (%)*
			RET			*NTRK3*		*BRAF*				*ALK*		*NTRK1*			*PPARG*					
**Fusion/SV driver**		**Total**	***CCDC6-RET***	***NCOA4-RET***	***RET-* Other**	***ETV6-NTRK3***	***NTRK3-* Other**	***AGK-BRAF***	***BRAF-SND1***	***BRAF* large deletion**	***BRAF-* Other**	***ALK-STRN***	***ALK-* Other**	***NTRK1-TPR***	***NTRK1-TPM3***	***NTRK1-* Other**	***PAX8-PPARG***	***CREB3L2-PPARG***	***LTK-***	***MET-***	***IGF2BP3***	***IGF2***
**EXPOSED INDIVIDUALS**																						
**Total**		**113 (100.0%)**	**19 (100.0%)**	**10 (100.0%)**	**11 (100.0%)**	**21 (100.0%)**	**2 (100.0%)**	**6 (100.0%)**	**3 (100.0%)**	**0**	**8 (100.0%)**	**6 (100.0%)**	**3 (100.0%)**	**4 (100.0%)**	**3 (100.0%)**	**4 (100.0%)**	**4 (100.0%)**	**3 (100.0%)**	**2 (100.0%)**	**1 (100.0%)**	**2 (100.0%)**	**1 (100.0%)**
# Breaks	Gain/loss at the breakpoint																					
2	<20 bp at both breakpoints	63 (55.8%)	11 (57.9%)	9 (90.0%)	5 (45.5%)	7 (33.3%)	0	6 (100.0%)	3 (100.0%)	0	5 (62.5%)	1 (16.7%)	1 (33.3%)	3 (75.0%)	3 (100.0%)	2 (50.0%)	2 (50.0%)	3 (100.0%)	0	1 (100.0%)	1 (50.0%)	0
2	<1000 bp at both breakpoints (20-999 bp at ≥1 breakpoint)	11 (9.7%)	1 (5.3%)	0	1 (9.1%)	3 (14.3%)	0	0	0	0	2 (25.0%)	0	0	0	0	1 (25.0%)	1 (25.0%)	0	1 (50.0%)	0	1 (50.0%)	0
2	≥ 1000 bp loss at ≥1 breakpoint	3 (2.7%)	0	0	1 (9.1%)	1 (4.8%)	0	0	0	0	0	0	0	0	0	0	1 (25.0%)	0	0	0	0	0
≥3	<20 bp at all breakpoints	1 (0.9%)	0	1 (10.0%)	0	0	0	0	0	0	0	0	0	0	0	0	0	0	0	0	0	0
≥3	<1000 bp at all breakpoints (20-999 bp at ≥1 breakpoint)	6 (5.3%)	1 (5.3%)	0	0	4 (19.0%)	0	0	0	0	1 (12.5%)	0	0	0	0	0	0	0	0	0	0	0
≥3	≥ 1000 bp loss at ≥1 breakpoint	20 (17.7%)	6 (31.6%)	0	4 (36.4%)	4 (19.0%)	1 (50.0%)	0	0	0	0	2 (33.3%)	2 (66.7%)	0	0	1 (25.0%)	0	0	0	0	0	0
Other special categories																						
Large deletion (≥ 1000 bp)		3 (2.7%)	0	0	0	0	0	0	0	0	0	3 (50.0%)	0	0	0	0	0	0	0	0	0	0
Large duplication (≥ 1000 bp; not tandem)		0	0	0	0	0	0	0	0	0	0	0	0	0	0	0	0	0	0	0	0	0
Tandem duplication (≥ 1000 bp)		0	0	0	0	0	0	0	0	0	0	0	0	0	0	0	0	0	0	0	0	0
Templated insertion		3 (2.7%)	0	0	0	2 (9.5%)	0	0	0	0	0	0	0	1 (25.0%)	0	0	0	0	0	0	0	0
Dicentric		2 (1.8%)	0	0	0	0	1 (50.0%)	0	0	0	0	0	0	0	0	0	0	0	1 (50.0%)	0	0	0
Chromothripsis		1 (0.9%)	0	0	0	0	0	0	0	0	0	0	0	0	0	0	0	0	0	0	0	1 (100.0%)
**UNEXPOSED INDIVIDUALS**																						
**Total**		**27 (100.0%)**	**6 (100.0%)**	**2 (100.0%)**	**4 (100.0%)**	**7 (100.0%)**	**1 (100.0%)**	**0**	**0**	**1 (100.0%)**	**1 (100.0%)**	**2 (100.0%)**	**0**	**0**	**0**	**0**	**0**	**1 (100.0%)**	**1 (100.0%)**	**0**	**1 (100.0%)**	**0**
# Breaks	Gain/loss at the breakpoint																					
2	<20 bp at both breakpoints	3 (11.1%)	1 (16.7%)	0	0	1 (14.3%)	1 (100.0%)	0	0	0	0	0	0	0	0	0	0	0	0	0	0	0
2	<1000 bp at both breakpoints (20-999 bp at ≥1 breakpoint)	3 (11.1%)	1 (16.7%)	0	0	1 (14.3%)	0	0	0	0	0	0	0	0	0	0	0	0	1 (100.0%)	0	0	0
2	≥ 1000 bp loss at ≥1 breakpoint	1 (3.7%)	0	0	0	1 (14.3%)	0	0	0	0	0	0	0	0	0	0	0	0	0	0	0	0
≥3	<20 bp at all breakpoints	0	0	0	0	0	0	0	0	0	0	0	0	0	0	0	0	0	0	0	0	0
≥3	<1000 bp at all breakpoints (20-999 bp at ≥1 breakpoint)	1 (3.7%)	0	0	1 (25.0%)	0	0	0	0	0	0	0	0	0	0	0	0	0	0	0	0	0
≥3	≥ 1000 bp loss at ≥1 breakpoint	12 (44.4%)	4 (66.7%)	2 (100.0%)	2 (50.0%)	3 (42.9%)	0	0	0	0	1 (100.0%)	0	0	0	0	0	0	0	0	0	0	0
Other special categories																						
Large deletion (≥ 1000 bp)		3 (11.1%)	0	0	0	0	0	0	0	1 (100.0%)	0	2 (100.0%)	0	0	0	0	0	0	0	0	0	0
Large duplication (≥ 1000 bp; not tandem)		3 (11.1%)	0	0	1 (25.0%)	1 (14.3%)	0	0	0	0	0	0	0	0	0	0	0	1 (100.0%)	0	0	0	0
Tandem duplication (≥ 1000 bp)		1 (3.7%)	0	0	0	0	0	0	0	0	0	0	0	0	0	0	0	0	0	0	1 (100.0%)	0
Templated insertion		0	0	0	0	0	0	0	0	0	0	0	0	0	0	0	0	0	0	0	0	0
Dicentric		0	0	0	0	0	0	0	0	0	0	0	0	0	0	0	0	0	0	0	0	0
Chromothripsis		0	0	0	0	0	0	0	0	0	0	0	0	0	0	0	0	0	0	0	0	0

**Mutation driver**	**Total**	***BRAF*** ^{V600E}	***BRAF* Other**	***NRAS***	***HRAS***	***KRAS***	***DICER1***	***TSHR***	***APC***	***NFE2L2***	***TSC2***
**EXPOSED INDIVIDUALS**											
**Total**	**172 (100.0%)**	**132 (100.0%)**	**4 (100.0%)**	**13 (100.0%)**	**11 (100.0%)**	**7 (100.0%)**	**0**	**2 (100.0%)**	**1 (100.0%)**	**1 (100.0%)**	**1 (100.0%)**
Single base substitution	163 (94.8%)	132 (100.0%)	2 (50.0%)	13 (100.0%)	10 (90.9%)	3 (42.9%)	0	2 (100.0%)	0	1 (100.0%)	0
Dinucleotide substitution	5 (2.9%)	0	0	0	1 (9.1%)	4 (57.1%)	0	0	0	0	0
Deletion	1 (0.6%)	0	1 (25.0%)	0	0	0	0	0	0	0	0
Insertion	1 (0.6%)	0	1 (25.0%)	0	0	0	0	0	0	0	0
Multiple mutations	2 (1.2%)	0	0	0	0	0	0	0	1 (100.0%)	0	1 (100.0%)
**UNEXPOSED INDIVIDUALS**											
**Total**	**43 (100.0%)**	**30 (100.0%)**	**0**	**4 (100.0%)**	**2 (100.0%)**	**2 (100.0%)**	**3 (100.0%)**	**1 (100.0%)**	**1 (100.0%)**	**0**	**0**
Single base substitution	37 (86.0%)	30 (100.0%)	0	4 (100.0%)	2 (100.0%)	0	0	1 (100.0%)	0	0	0
Dinucleotide substitution	2 (4.7%)	0	0	0	0	2 (100.0%)	0	0	0	0	0
Deletion	0	0	0	0	0	0	0	0	0	0	0
Insertion	0	0	0	0	0	0	0	0	0	0	0
Multiple mutations	4 (9.3%)	0	0	0	0	0	3 (100.0%)	0	1 (100.0%)	0	0

Abbreviations: basepair (bp), papillary thyroid carcinoma (PTC), structural variant (SV).

* Column percent for fusion/SV and mutation drivers separately.

Supplementary Table 2. Distribution of 69 fusion/SV-driven PTCs from TCGA by the pattern of DNA damage that generated the driver, by driver gene and fusion partner

**Driver type** **Detailed classification**		N (%)	N (%)	N (%)	N (%)	N (%)	N (%)	N (%)	N (%)	N (%)	N (%)	N (%)	N (%)	N (%)
			RET			***NTRK3***								
		**Total**	***CCDC6-RET***	***NCOA4-RET***	***RET*-Other**	***ETV6-NTRK3***	***NTRK3-RBPMS***	***BRAF-***	***ALK-***	***NTRK1-***	***PAX8-PPARG***	***MET-***	***FGFR2-***	***IGF2BP3***
Total		69 (100.0%)	20 (100.0%)	5 (100.0%)	6 (100.0%)	5 (100.0%)	1 (100.0%)	12 (100.0%)	4 (100.0%)	3 (100.0%)	4 (100.0%)	1 (100.0%)	2 (100.0%)	6 (100.0%)
# Breaks	Gain/loss at the breakpoint													
2	<20 bp at both breakpoints	17 (24.6%)	6 (30.0%)	2 (40.0%)	3 (50.0%)	0	0	3 (25.0%)	1 (25.0%)	0	1 (25.0%)	0	0	1 (16.7%)
2	<1000 bp at both breakpoints (20-999 bp at ≥1 breakpoint)	13 (18.8%)	4 (20.0%)	1 (20.0%)	0	1 (20.0%)	1 (100.0%)	2 (16.7%)	0	0	1 (25.0%)	0	1 (50.0%)	2 (33.3%)
2	≥ 1000 bp loss at ≥1 breakpoint	3 (4.3%)	0	0	0	0	0	1 (8.3%)	0	0	1 (25.0%)	1 (100.0%)	0	0
≥3	<20 bp at all breakpoints	1 (1.4%)	1 (5.0%)	0	0	0	0	0	0	0	0	0	0	0
≥3	<1000 bp at all breakpoints (20-999 bp at ≥1 breakpoint)	7 (10.1%)	2 (10.0%)	0	1 (16.7%)	1 (20.0%)	0	1 (8.3%)	0	0	0	0	0	2 (33.3%)
≥3	≥ 1000 bp loss at ≥1 breakpoint	24 (34.8%)	7 (35.0%)	2 (40.0%)	2 (33.3%)	3 (60.0%)	0	3 (25.0%)	2 (50.0%)	3 (100.0%)	1 (25.0%)	0	0	1 (16.7%)
Other special categories														
Large deletion (≥ 1000 bp)		1 (1.4%)	0	0	0	0	0	0	1 (25.0%)	0	0	0	0	0
Large duplication (≥ 1000 bp; not tandem)		1 (1.4%)	0	0	0	0	0	0	0	0	0	0	1 (50.0%)	0
Tandem duplication (≥ 1000 bp)		2 (2.9%)	0	0	0	0	0	2 (16.7%)	0	0	0	0	0	0

Abbreviations: basepair (bp), papillary thyroid carcinoma (PTC), structural variant (SV).

Supplementary Table 3. Distribution of inversions versus translocations for PTCs with fusion/SV drivers generated from 2 DNA DSBs (N=84 from the Chornobyl Tissue Bank, N=33 from TCGA)

			RET			*NTRK3*		*BRAF*			*ALK*		*NTRK1*			*PPARG*					
		Total	*CCDC6-RET*	*NCOA4-RET*	*RET*- Other	*ETV6-NTRK3*	*NTRK3*-Other	*AGK-BRAF*	*BRAF-SND1*	*BRAF*-Other	*ALK-STRN*	*ALK*- Other	*NTRK1-TPR*	*NTRK1-TPM3*	*NTRK1*-Other	*PAX8-PPARG*	*CREB3L2-PPARG*	*LTK*-	*MET*-	*IGF2BP3*	*FGFR2*-
**Fusion/SV driver**		**N (%)***	**N (%)***	**N (%)***	**N (%)***	**N (%)***	**N (%)***	**N (%)***	**N (%)***	**N (%)***	**N (%)***	**N (%)***	**N (%)***	**N (%)***	**N (%)***	**N (%)***	**N (%)***	**N (%)***	**N (%)***	**N (%)***	**N (%)***
**EXPOSED INDIVIDUALS**																					
**Total**		**77**	**12**	**9**	**7**	**11**	**0**	**6**	**3**	**7**	**1**	**1**	**3**	**3**	**3**	**4**	**3**	**1**	**1**	**2**	**0**
# Breaks	Gain/loss at the breakpoint																				
2	<20 bp at both breakpoints																				
	Inversions	42 (66.7%)	11 (100.0%)	9 (100.0%)	1 (20.0%)	0	0	6 (100.0%)	3 (100.0%)	3 (60.0%)	0	1 (100.0%)	3 (100.0%)	3 (100.0%)	1 (50.0%)	0	0	0	1 (100.0%)	0	0
	Translocations	21 (33.3%)	0 (0.0%)	0 (0.0%)	4 (80.0%)	7 (100.0%)	0 #DIV/0!	0	0	2 (40.0%)	1 (100.0%)	0	0	0	1 (50.0%)	2 (100.0%)	3 (100.0%)	0	0	1 (100.0%)	0
2	<1000 bp at both breakpoints (20-999 bp at ≥1 breakpoint)																				
	Inversions	2 (18.2%)	1 (100.0%)	0	0	0	0	0	0	0	0	0	0	0	1 (100.0%)	0	0	0	0	0	0
	Translocations	9 (81.8%)	0	0	1 (100.0%)	3 (100.0%)	0	0	0	2 (100.0%)	0	0	0	0	0 (0.0%)	1 (100.0%)	0	1 (100.0%)	0	1 (100.0%)	0
2	≥ 1000 bp loss at ≥1 breakpoint																				
	Inversions	0	0	0	0	0	0	0	0	0	0	0	0	0	0	0	0	0	0	0	0
	Translocations	3 (100.0%)	0	0	1 (100.0%)	1 (100.0%)	0	0	0	0	0	0	0	0	0	1 (100.0%)	0	0	0	0	0
**UNEXPOSED INDIVIDUALS**																					
**Total**		**7**	**2**	**0**	**0**	**3**	**1**	**0**	**0**	**0**	**0**	**0**	**0**	**0**	**0**	**0**	**0**	**1**	**0**	**0**	**0**
# Breaks	Gain/loss at the breakpoint																				
2	<20 bp at both breakpoints																				
	Inversions	1 (33.3%)	1 (100.0%)	0	0	0	0	0	0	0	0	0	0	0	0	0	0	0	0	0	0
	Translocations	2 (66.7%)	0	0	0	1 (100.0%)	1 (100.0%)	0	0	0	0	0	0	0	0	0	0	0	0	0	0
2	<1000 bp at both breakpoints (20-999 bp at ≥1 breakpoint)																				
	Inversions	1 (33.3%)	1 (100.0%)	0	0	0	0	0	0	0	0	0	0	0	0	0	0	0	0	0	0
	Translocations	2 (66.7%)	0	0	0	1 (100.0%)	0	0	0	0	0	0	0	0	0	0	0	1 (100.0%)	0	0	0
2	≥ 1000 bp loss at ≥1 breakpoint																				
	Inversions	0	0	0	0	0	0	0	0	0	0	0	0	0	0	0	0	0	0	0	0
	Translocations	1 (100.0%)	0	0	0	1 (100.0%)	0	0	0	0	0	0	0	0	0	0	0	0	0	0	0
**TCGA**																					
**Total**		**33**	**10**	**3**	**3**	**1**	**1**	**1**	**1**	**4**	**0**	**1**	**0**	**0**	**0**	**3**	**0**	**0**	**1**	**3**	**1**
# Breaks	Gain/loss at the breakpoint																				
2	<20 bp at both breakpoints																				
	Inversions	9 (52.9%)	6 (100.0%)	2 (100.0%)	0	0	0	1 (100.0%)	0	0	0	0	0	0	0	0	0	0	0	0	0
	Translocations	8 (47.1%)	0	0	3 (100.0%)	0	0	0	0	2 (100.0%)	0	1 (100.0%)	0	0	0	1 (100.0%)	0	0	0	1 (100.0%)	0
2	<1000 bp at both breakpoints (20-999 bp at ≥1 breakpoint)																				
	Inversions	7 (53.8%)	4 (100.0%)	1 (100.0%)	0	0	0	0	1 (100.0%)	0	0	0	0	0	0	0	0	0	0	0	1 (100.0%)
	Translocations	6 (46.2%)	0	0	0	1 (100.0%)	1 (100.0%)	0	0	1 (100.0%)	0	0	0	0	0	1 (100.0%)	0	0	0	2 (100.0%)	0
2	≥ 1000 bp loss at ≥1 breakpoint																				
	Inversions	1 (33.3%)	0	0	0	0	0	0	0	1 (100.0%)	0	0	0	0	0	0	0	0	0	0	0
	Translocations	2 (66.7%)	0	0	0	0	0	0	0	0	0	0	0	0	0	1 (100.0%)	0	0	1 (100.0%)	0	0

Abbreviations: basepair (bp), papillary thyroid carcinoma (PTC), structural variant (SV).

Supplementary Table 4. Distribution of PTCs by the pattern of DNA damage that generated the driver, by radiation dose

		Unexposed	Exposed, overall and by dose (mGy)						
**Driver type**		**Total**	**Total**	**1-<100**	**100-<200**	**200-<500**	**≥500**		
**Detailed classification**		**N (%)***	**N (%)***	**N (%)***	**N (%)***	**N (%)***	**N (%)***	**Median (mGy)†**	**Mean (mGy)†**
**Total**		70 (100.0%)	285 (100.0%)	153 (100.0%)	66 (100.0%)	36 (100.0%)	30 (100.0%)	58	147
**Fusion/SV**									
# Breaks	Gain/loss at the breakpoint								
2	<20 bp at both breakpoints	3 (11.1%)	63 (55.8%)	15 (36.6%)	14 (50.0%)	20 (90.9%)	14 (63.6%)	208	315
	Inversions	1 (3.7%)	42 (37.2%)	5 (12.2%)	11 (39.3%)	16 (72.7%)	10 (45.5%)	233	345
	Non-*RET-*	0 (0.0%)	21 (18.6%)	0 (0.0%)	7 (25.0%)	11 (50.0%)	3 (13.6%)	245	330
	RET-	1 (3.7%)	21 (18.6%)	5 (12.2%)	4 (14.3%)	5 (22.7%)	7 (31.8%)	222	360
	Translocations	2 (7.4%)	21 (18.6%)	10 (24.4%)	3 (10.7%)	4 (18.2%)	4 (18.2%)	58	259
	Non-*RET-*	2 (7.4%)	17 (15.0%)	8 (19.5%)	2 (7.1%)	3 (13.6%)	4 (18.2%)	55	289
	RET-	0 (0.0%)	4 (3.5%)	2 (4.9%)	1 (3.6%)	1 (4.5%)	0 (0.0%)	106	117
2	<1000 bp at both breakpoints (20-999 bp at ≥1 breakpoint)	3 (11.1%)	11 (9.7%)	3 (7.3%)	5 (17.9%)	1 (4.5%)	2 (9.1%)	131	187
2	≥ 1000 bp loss at ≥1 breakpoint	1 (3.7%)	3 (2.7%)	2 (4.9%)	1 (3.6%)	0	0 (0.0%)	38	47
≥3	<20 bp at all breakpoints	0	1 (0.9%)	1 (2.4%)	0	0	0 (0.0%)	†	†
≥3	<1000 bp at all breakpoints (20-999 bp at ≥1 breakpoint)	1 (3.7%)	6 (5.3%)	2 (4.9%)	2 (7.1%)	0	2 (9.1%)	120	268
≥3	≥ 1000 bp loss at ≥1 breakpoint	12 (44.4%)	20 (17.7%)	15 (36.6%)	4 (14.3%)	0	1 (4.5%)	36	62
Other special categories									
Large deletion (≥ 1000 bp)		3 (11.1%)	3 (2.7%)	1 (2.4%)	1 (3.6%)	0	1 (4.5%)	18	195
Large duplication (≥ 1000 bp; not tandem)		3 (11.1%)	0	0	0	0	0	0	0
Tandem duplication (≥ 1000 bp)		1 (3.7%)	0	0	0	0	0	†	†
Templated insertion		0	3 (2.7%)	1 (2.4%)	1 (3.6%)	0	1 (4.5%)	188	271
Dicentric		0	2 (1.8%)	1 (2.4%)	0	1 (0.0%)	0	†	†
Chromothripsis		0	1 (0.9%)	0	0	0	1 (4.5%)	†	†
**Mutation (by type)**									
Single base substitution		37 (86.0%)	163 (94.8%)	106 (94.6%)	38 (100.0%)	13 (92.9%)	6 (75.0%)	49	95
Dinucleotide substitution		2 (4.7%)	5 (2.9%)	4 (3.6%)	0	0	1 (12.5%)	28	167
Deletion		0	1 (0.6%)	1 (0.9%)	0	0	0	†	†
Insertion		0	1 (0.6%)	1 (0.9%)	0	0	0	†	†
Multiple mutations		4 (9.3%)	2 (1.2%)	0	0	1 (7.1%)	1 (12.5%)	0	182
**Mutation (by major category)**									
$BRAF^{V600E}$		30 (69.8%)	132 (76.7%)	88 (78.6%)	28 (73.7%)	11 (78.6%)	5 (62.5%)	49	94
RAS		8 (18.6%)	31 (18.0%)	21 (18.8%)	8 (21.1%)	1 (7.1%)	1 (12.5%)	41	86

Abbreviations:

* Column percent of fusion and mutation drivers separately.

† Mean and median are provided only for groups with >2 individuals.

Supplementary Table 5. Relationship of radiation dose to the thyroid* with DNA DSBs and *CLIP2* expression, by pattern of DNA damage that generated the PTC driver

		Adjusted for sex	
Driver category	N	β per 100 mGy (95%CI)†	P
<u>**Clonal deletion:SNV ratio (>=90)**</u>			
2 breaks, <20 bp at both breakpoints	63	0.0061 (0.0028 , 0.0094)	5.1E-04
Inversions	42	0.0075 (0.0027 , 0.012)	0.0031
Non-*RET* -	21	0.0042 (-0.00070 , 0.0090)	0.086
RET -	21	0.013 (0.0025 , 0.024)	0.017
Translocations	21	0.0032 (0.00015 , 0.0063)	0.039
Non-*RET* -	17	0.0031 (-0.00038 , 0.0066)	0.072
3 breaks, ≥ 1000 bp loss at ≥1 breakpoint	20	-0.0043 (-0.014 , 0.0052)	0.27
BRAF ^{V600E}	132	0.0043 (-0.0014 , 0.010)	0.14
RAS mutation	31	0.0087 (-0.0011 , 0.019)	0.080
<u>***CLIP2* expression**</u>			
2 breaks, <20 bp at both breakpoints	63	0.016 (-0.020 , 0.053)	0.37
Inversions	42	0.014 (-0.032 , 0.059)	0.55
Non-*RET* -	21	0.010 (-0.038 , 0.057)	0.67
RET -	21	0.007 (-0.087 , 0.10)	0.88
Translocations	21	0.019 (-0.048 , 0.085)	0.56
Non-*RET* -	17	0.028 (-0.029 , 0.085)	0.31
3 breaks, ≥ 1000 bp loss at ≥1 breakpoint	20	-0.36 (-0.98 , 0.25)	0.17
BRAF ^{V600E}	132	-0.012 (-0.15 , 0.13)	0.86
RAS mutation	31	-0.017 (-0.31 , 0.27)	0.91

* Analyses excluded unexposed individuals (i.e., born >9 months after the accident)

† Estimates from linear regression models predicting each molecular characteristic. Models were adjusted for sex and age at PTC. Radiation dose outliers were truncated at 1000 mGy for PTC with fusion drivers with 2 breaks, <20 bp at both breakpoints and 300 mGy for all other analyses. Note that P=0.039 if dose was also truncated at 300 mGy for PTC with fusion drivers with 2 breaks.

Supplementary Table 6. Relationship between clonal clock mutations (SBS1, SBS5) and age at the time of the Chornobyl accident*, by pattern of DNA damage that generated the PTC driver

		SBS1+SBS5			SBS1			SBS5		
**Driver category**	**N**	**β per year of age (95%CI)†**		**P**	**β per year of age (95%CI)†**		**P**	**β per year of age (95%CI)†**		**P**
2 breaks, <20 bp at both breakpoints	63	10.7	(4.6 , 16.8)	8.2E-04	1.5	(0.6 , 2.3)	8.5E-04	9.5	(3.9 , 15.2)	1.2E-03
Inversions	42	11.4	(3.5 , 19.3)	0.0055	2.0	(1.1 , 2.9)	5.02E-05	9.4	(2.2 , 16.7)	0.0121
Non-*RET* -	21	11.3	(4.5 , 18.2)	0.0023	2.0	(1.2 , 2.8)	3.07E-05	9.3	(2.5 , 16.0)	0.0089
RET -	21	18.9	(2.0 , 35.9)	0.029	2.8	(1.0 , 4.5)	0.003	16.2	(0.4 , 32.0)	0.043
Translocations	21	6.8	(-6.8 , 20.4)	0.30	-0.1	-(2.0 , 1.9)	0.94	7.2	-(5.7 , 20.1)	0.26
Non-*RET* -	17	6.0	(-11.4 , 23.4)	0.47	-0.1	-(2.7 , 2.4)	0.92	6.4	-(10.1 , 22.9)	0.42
RET -	4	~								
3 breaks, ≥ 1000 bp loss at ≥1 breakpoint	20	7.3	(-4.6 , 19.1)	0.21	0.2	-(1.2 , 1.6)	0.74	7.0	-(4.0 , 18.1)	0.19
$BRAF^{V600E}$	132	-2.8	(-7.3 , 1.7)	0.22	0.1	-(0.5 , 0.6)	0.79	-2.9	-(7.1 , 1.2)	0.17
RAS mutation	31	-1.8	(-12.8 , 9.3)	0.74	-0.3	-(2.1 , 1.4)	0.69	-1.4	-(11.4 , 8.5)	0.77

* Analyses excluded unexposed individuals (i.e., born >9 months after the accident)

† Estimates from linear regression models predicting clonal clock mutations with adjustment for sex and age at PTC; outliers with >500 clonal clock mutations were truncated at 500.

Supplementary Data S1. Driver breakpoint characterizations for samples included in this analysis from both Chornobyl and TCGA

Sample: Sample ID (Chornobyl sample IDs begin with the four letters "REBC," TCGA sample IDs begin with the four letters "TCGA"

Designated_Driver: Oncogenic driver gene (and fusion partner for fusion drivers)

DetailedDriverType: Mutation type or breakpoint characterization for fusion drivers

Inversion_Translocation: Specification of whether simple fusion drivers were inversions or translocations
